# EpiScan: accurate high-throughput mapping of antibody-specific epitopes using sequence information

**DOI:** 10.1038/s41540-024-00432-7

**Published:** 2024-09-09

**Authors:** Chuan Wang, Jiangyuan Wang, Wenjun Song, Guanzheng Luo, Taijiao Jiang

**Affiliations:** 1 School of Life Sciences, Sun Yat-sen University, Guangzhou, China; 2 Guangzhou National Laboratory, Guangzhou, China; 3 Institute of Integration of Traditional and Western Medicine, The First Affiliated Hospital of Guangzhou Medical University, Guangzhou, China; 4 State Key Laboratory of Respiratory Disease, The Key laboratory of Advanced Interdisciplinary Studies Center, the First Affiliated Hospital of Guangzhou Medical University, Guangzhou, China

**Keywords:** Programming language, Protein design

## Abstract

The identification of antibody-specific epitopes on virus proteins is crucial for vaccine development and drug design. Nonetheless, traditional wet-lab approaches for the identification of epitopes are both costly and labor-intensive, underscoring the need for the development of efficient and cost-effective computational tools. Here, EpiScan, an attention-based deep learning framework for predicting antibody-specific epitopes, is presented. EpiScan adopts a multi-input and single-output strategy by designing independent blocks for different parts of antibodies, including variable heavy chain (V_H_), variable light chain (V_L_), complementary determining regions (CDRs), and framework regions (FRs). The block predictions are weighted and integrated for the prediction of potential epitopes. Using multiple experimental data samples, we show that EpiScan, which only uses antibody sequence information, can accurately map epitopes on specific antigen structures. The antibody-specific epitopes on the receptor binding domain (RBD) of SARS coronavirus 2 (SARS-CoV-2) were located by EpiScan, and the potentially valuable vaccine epitope was identified. EpiScan can expedite the epitope mapping process for high-throughput antibody sequencing data, supporting vaccine design and drug development. Availability: For the convenience of related wet-experimental researchers, the source code and web server of EpiScan are publicly available at https://github.com/gzBiomedical/EpiScan.

## Introduction

The humoral immune system defends against foreign bodies, such as bacteria and viruses, by producing B cells that generate antibodies^1^. Antibodies recognize and bind to pathogenic substances called antigens, playing a critical role in the immune response. B cell epitopes (BCEs) are specific regions on the surface of antigens that induce humoral immunity by being recognized by B cell receptors or specific antibodies. Identifying BCEs can aid in designing effective vaccines, thus contributing to disease prevention and diagnosis^2^.

The traditional methods for identifying antigenic epitopes primarily involve chemical or biological techniques that accurately identify epitope residues. Experimental structure determination methods, such as X-ray crystallography, nuclear magnetic resonance spectroscopy, and cold electron microscopy, provide the gold standards for characterizing antibody–antigen binding patterns^3^. However, experimental-based methods are labor-intensive and costly, making them challenging to use on a large scale. Alternatively, computational models can be built to identify antigen epitopes as candidate epitopes, which can then be screened by experiment to identify real epitopes. The development of highly accurate BCE prediction models has become a rapidly growing area of research due to their increased efficiency and cost-effectiveness^4,5^

BCEs can be divided into linear epitopes and conformational epitopes based on their structures. Many methods, such as support vector machines^6^ and random forests, predict linear epitopes with amino-acid sequences^7^. However, most BCEs are conformational, i.e., they are distal in the sequence but close in 3D structure^2^. Several methods, including BeTop^8^, PAIRPred^9^, and DiscoTope-2.0^10^, apply neural network methods to the structural characteristics of antigen residues. Some of these methods improve their performance by introducing new features, such as statistical features in BeTop, thick surface slices in PAIRPred, and new spatial neighborhood definitions and hemispheric exposure in DiscoTope-2.0. Despite these methodologies, their efficacy in forecasting B-cell conformational epitopes remains limited^11^. Furthermore, the aforementioned studies failed to incorporate information specific to antibodies. In fact, some literature suggests that antibody-agnostic epitope prediction constitutes a poorly delineated challenge^12,13^ given that epitopes only attain functionality within the framework of their corresponding antibody^14^. Recent studies have developed new methods that predict the epitope for a specific antibody by utilizing the information available within the sequence or structure of the antibody^15^. The EpiPred method utilizes information about antigen-antibody interaction to achieve the most advanced performance at the time of its development^16^. PECAN^17^ and MaSIF^18^ are recent structure-based BCE prediction methods that use graph convolution operations to learn and utilize geometric features. A notable detail is that due to the concentration of the main differences between antibodies in the variable region, particularly the third complementarity-determining regions (CDR3) loops of the heavy chain, some methods only model the antibody variable region to predict specific binding epitopes^17,19^ However, predicting the structure and function of CDR3 is notoriously difficult. Moreover, many methods simulate the entire antibody region as a single structure without considering the interrelationship among different parts within the antibody^19–22^ Nevertheless, antibody-specific binding sites have notably different weight distributions in V_L_, V_H,_ and different CDRs^23,24^ The CDRs and FRs are critical in preserving the conformation of antibody-specific binding^25,26^ Therefore, functional modeling of different antibody regions is a strategy that aligns closely with biological principles, and it has the potential to improve the accuracy of antibody-specific epitope mapping^27^.

In this work, a unified deep learning-based framework, EpiScan, was proposed for predicting antibody-specific BCEs. EpiScan adopts a multi-input and single-output (MISO) strategy^28^. Independent submodels of V_L_, V_H_, CDRs, and FRs of antibodies were designed to capture their different roles in antibody-antigen binding. The structure information of antigens and antibodies was inputted into different modules for processing. The output of these sub-models was weighted by the attention-scoring function. The weighted results were then merged into a fusion model to generate the final prediction of BCEs. The use of attention modules in MISO tasks has been shown to well capture the coupling relationship between the components of the model in previous studies^29–31^ In addition, a protein large language model was employed to encode the antibody sequence as an “embedded” vector, serving as the model’s input, to further characterize the antibody sequence. The experimental data were used to train and verify EpiScan, and the results showed that the model trained on the proposed framework can overcome the limitations of current computing methods and achieve the most advanced performance in epitope prediction tasks. EpiScan showed significant improvement in predicting antibody-specific epitopes compared with the existing methods for predicting BCEs. Furthermore, the performance of EpiScan in different antigen and antibody types was evaluated, and its effectiveness in a wide range of application scenarios was confirmed. EpiScan’s flexibility allows for customization for a specific antibody–antigen binding system to improve the prediction accuracy, and it can be easily combined with other bioinformatics methods to provide a more comprehensive immunoassay.

## Results

### Motivation and key idea

The rapid growth of high-throughput antibody sequencing data has opened up new possibilities for vaccine development. Identifying highly immunogenic epitopes is crucial for designing effective vaccines, and we hypothesize that these epitopes are likely to be frequently targeted by neutralizing antibodies. Similar antibodies may target two completely different conformational regions of the same antigen, which makes the epitope mapping highly challenging. To address this challenge, we developed EpiScan, incorporating biological principles into its model framework. It employs an attention mechanism within a neural network to model antibody internal dynamics and capture the underlying logic of epitope recognition. Additionally, EpiScan employs a protein language model to efficiently encode antibody amino acid sequences, thereby facilitating the retrieval of pertinent information solely from sequence data. We believe that the modeling paradigm employed by EpiScan holds great promise for addressing the challenges associated with epitope mapping against a specific antigen using high-throughput antibody sequences.

### Overview of EpiScan

**Fig. 1:**
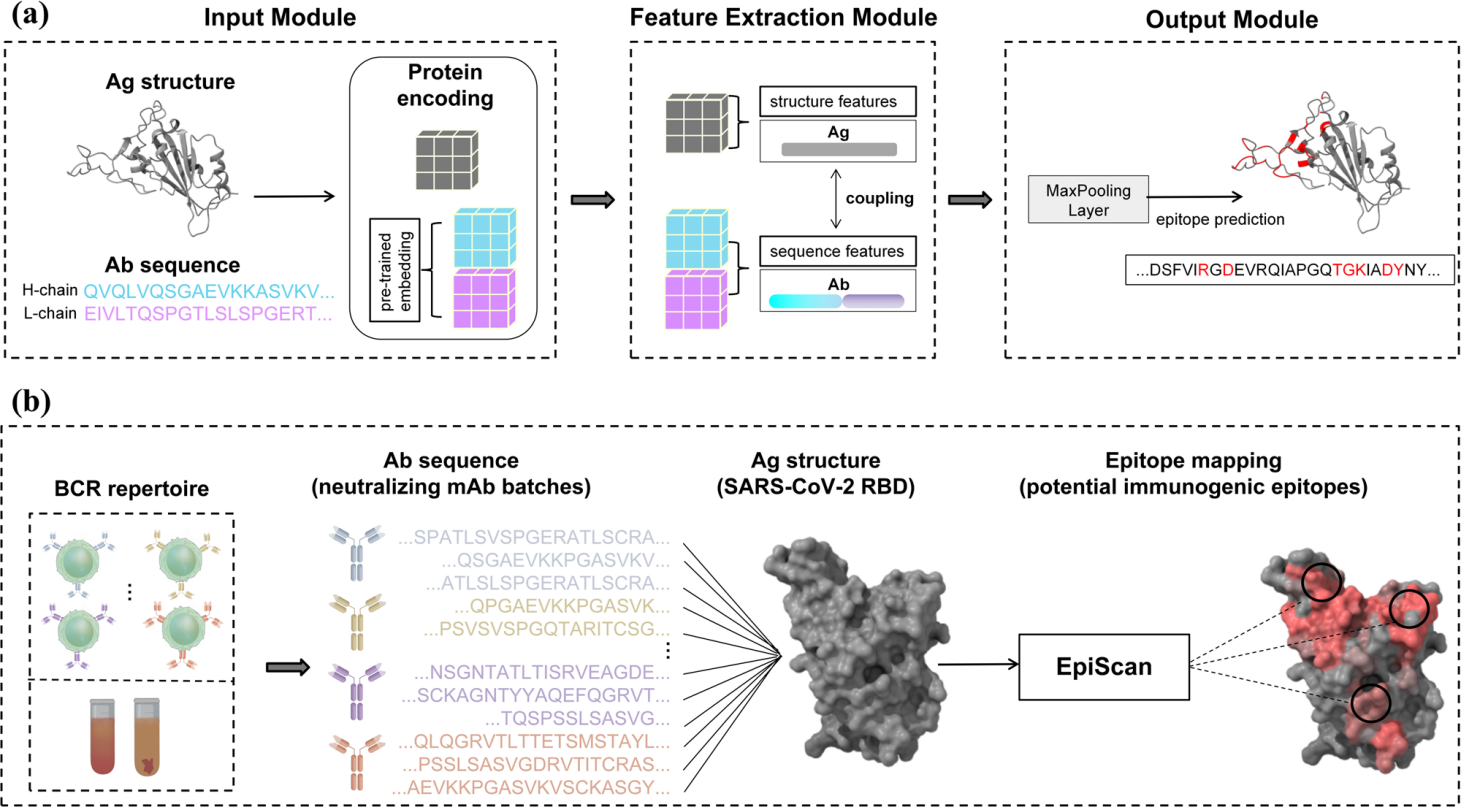
Motivation and the overall architecture of EpiScan. **a** The EpiScan framework consists of three primary modules: (1) Input module, which incorporates a matrix representation of antigen-antibody pairs. These matrix input pairs are processed by the encoding layer, where the antibody sequence is encoded using a pre-trained deep learning language model (Bepler and Berger); (2) Feature extraction module, comprising four distinct blocks: Binding, Hinge, Rotation, and ECA. The pre-encoded matrix undergoes feature extraction to obtain antigen structural features and antibody sequence features. Each block implements the simulation of antigen-antibody coupling, resulting in an output probability matrix corresponding to the epitope length of the antigen primary structure; (3) Output module, designed to predict unknown interactions within antibody-antigen pairs and capable of addressing regression tasks. **b** Workflow for mapping potential immunogenic epitopes, demonstrated in a case study using the SARS-CoV-2 RBD structure: High-throughput neutralizing antibody sequencing data from sera of naturally infected or vaccinated survivors are used to batch-map the epitopes of each neutralizing antibody on a specific antigen structure. This process identifies immunologically advantageous regions, providing valuable insights for vaccine and drug design.

We present the motivation and the overall architecture of EpiScan, as illustrated in Fig. 1. EpiScan is a comprehensive framework composed of three primary modules that work together to predict unknown interactions within antibody-antigen pairs. In the following subsections, we describe the materials and methods used in each of the three primary modules of the EpiScan framework in detail.

The EpiScan framework consists of an Input module, Feature extraction module, and Output module. Given two input matrices(Ag-Ab pairs), antigen ${Z}_{{Ag}}={\{{z}_{{{ag}}_{i}}\}}_{i=1}^{{L}_{{Ag}}}$ and antibody ${Z}_{{Ab}}={\{{z}_{{{ab}}_{i}}\}}_{i=1}^{{L}_{{Ab}}}$, the network assigns output ${Z}_{{out}}$ to each sample point ${p}_{i}\in P$ a probability of belonging to the positive class (i.e.binding amino-acid residue). The set of sample points $P$ refers explicitly to the individual residues of the Ag that are considered in epitope mapping analysis. Each antigenic amino acid is treated as a distinct sample point for the purpose of feature extraction and epitope prediction.

The input module of EpiScan consists of several layers that process the input matrix of the antigen and antibody. First, the input data is processed by an embedding block, which converts each amino acid in the sequence into a high-dimensional representation. The pre-trained layer only processes the antibody sequence and is not fine-tuned during training. The output of the pre-trained layer is passed through a linear layer, a ReLU layer, and a dropout layer, which help to reduce overfitting and improve the performance of the model. The output module of EpiScan consists of a max-pooling layer, which is applied to the output of the input module to reduce the dimensionality of the features and extract the most important information. The resulting feature vector is then passed through the epitope prediction(sigmoid) layer, which predicts the likelihood of each position in the antigen sequence being part of an epitope. The EpiScan is trained using a specific loss function (Section “Methods” details-Cost and optimizer function), and the predicted epitope residues are marked as positive samples.

### Evaluation of the performance of EpiScan on DB1 dataset

As shown in Table 1, a comparison of baseline methods for predicting antibody-specific epitopes on DB1 is presented in terms of ${Precision}$, ${Recall}$, $F1{\_score}$, ${MCC}$, ${AUROC}$, and ${AU}{PR}$. EpiScan, the proposed SOTA model, achieved the best overall performance among all the methods. In terms of ${Precision}$, EpiScan achieved a value of 0.239 ± 0.019, outperforming most methods except for DeepBindPPI with a ${Precision}$ of 0.315. EpiScan also exhibited a slightly higher ${Recall}$ of 0.776 ± 0.038 compared to PInet, the method with the next highest ${Recall}$ of 0.774. Remarkably, EpiScan attained the highest $F1{\_score}$ of 0.338 ± 0.021, surpassing the $F1{\_score}$ of other models by a significant margin. The exact $F1{\_score}$ value for PInet is not provided in the table for comparison. Additionally, EpiScan achieved the best ${AU}{ROC}$ of 0.715 ± 0.008, which was 0.5% higher than the second-best performing method, EPI-EPMP, with an ${AU}{ROC}$ of 0.710 ± 0.003.

**Table 1:** A comparison of baseline methods for predicting antibody-specific epitopes on DB1 ( ± std) is presented in terms of *Precision*, *Recall*, *F1_score*, the area under the receiver operating characteristic curve (*AUROC*), and the area under the precision-recall curve (*AUPR*)

Input		Methods	*Precision*↑	*Recall*↑	*AUROC*↑	*AUPR*↑	*F1_score*↑	*MCC*↑
Ab	Ag							
struct	struct	EPI-EPMP (2021)^19^	NA	NA	0.710 ± 0.003	0.277 ± 0.002	NA	NA
		EPI-CNN-GCN (2021)^19^	NA	NA	0.634 ± 0.003	0.210 ± 0.001	NA	NA
		EpiPred (2014)^16^	0.136	0.436	NA	NA	0.204	0.156
		DiscoTope (2012)^10^	0.214	0.110	NA	NA	0.051	0.096
		Sppider (2007)^11^	0.153	0.363	NA	NA	NA	NA
		AbAdapt (2022)^20^	0.156	0.676	NA	0.243	NA	NA
		DeepBindPPI (2023)^21^	**0.315**	0.413	0.665	NA	NA	NA
		PECAN (2020)^17^	0.157	0.730	0.655	0.226	NA	NA
		PInet (2021)^58^	0.216	0.774	0.687	**0.368**	NA	NA
seq	struct	**EpiScan**	0.239 ± 0.019	**0.776** ± **0.038**	**0.715** ± **0.008**	0.304 ± 0.009	**0.338** ± **0.021**	**0.275** ± **0.018**

Bold values indicate the best performance for each metric across methods.

These results indicate that the EpiScan model excels in predicting antibody-specific epitopes on the DB1 dataset. The model’s multi-feature representation of proteins and its attention to finer granularity information appear to be beneficial for accurate prediction. Notably, EpiScan outperformed the best-performing comparison model, PInet, in the ${AU}{ROC}$, ${AU}{PR}$, and $F1{\_score}$ also ${MCC}$ metrics, demonstrating its effectiveness as a SOTA model.

### Evaluation of the performance of EpiScan on DB2 dataset

The generalization of EpiScan was evaluated with a separate test set (DB2). As shown in Table 2, EpiScan outperformed state-of-the-art methods including PInet across all evaluated metrics. Specifically, it achieved a Precision of 0.215, significantly higher than DeepBindPPI(0.201). Moreover, EpiScan demonstrated an improved Recall of 0.855 compared to PInet’s 0.825. In terms of ${AUROC}$, EpiScan also showed enhancement with a score of 0.686, versus 0.647 for AbAdapt. Similarly, EpiScan obtained a higher ${AUPR}$ of 0.243 than PInet’s 0.168. Lastly, EpiScan had a superior $F1{\_score}$ of 0.327, whereas PInet reached only 0.238. In addition, EpiScan achieved a ${MCC}$ of 0.264, surpassing PInet’s 0.228. Compared to other recent methods like DeepBindPPI, AbAdapt, and EPI-CNN-GCN, EpiScan showed consistently stronger performance, highlighting its state-of-the-art epitope prediction capabilities generalizable across datasets.

**Table 2:** Comparison of EpiScan with the state-of-the-art method on DB2

Methods	*Precision*↑	*Recall*↑	*AUROC*↑	*AUPR*↑	*F1-score*↑	*MCC*↑
PInet (2021)	0.146	0.825	0.562	0.168	0.238	0.158
DeepBindPPI (2023)	0.116	0.627	0.528	0.118	0.184	0.079
AbAdapt (2022)	0.134	0.604	0.527	0.137	0.203	0.101
EPI-CNN-GCN (2021)	0.191	0.406	0.509	0.119	0.190	0.077
EpiPred (2014)	0.072	0.058	NA	NA	0.045	−0.005
**EpiScan**	**0.215**	**0.855**	**0.686**	**0.243**	**0.327**	**0.264**

Bold values indicate the best performance for each metric across methods.

**Fig. 2:**
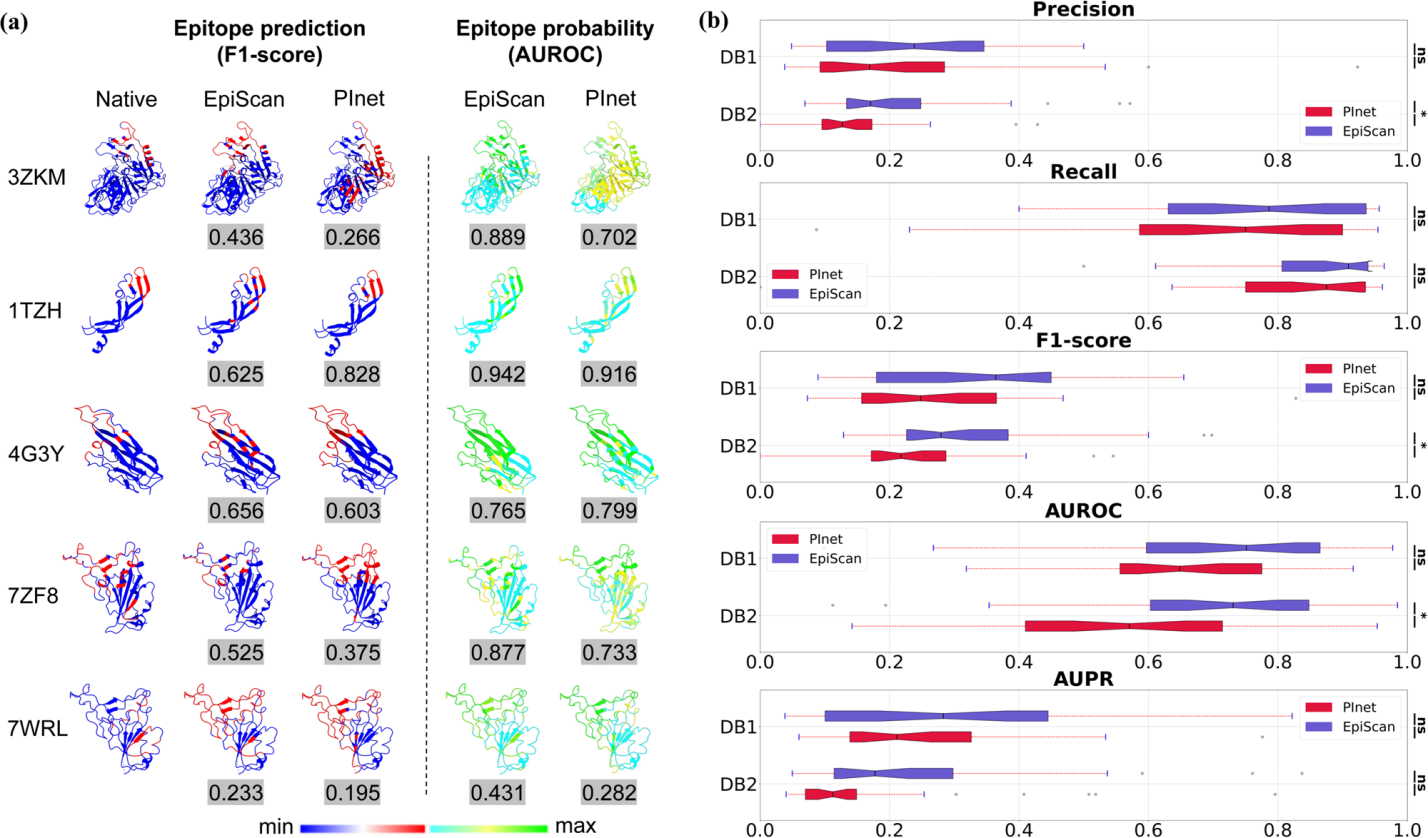
Comparison of epitope prediction and performance evaluation of EpiScan and PInet on DB1 and DB2 datasets. **a** Epitope map visualization for representative queries, depicting native epitopes (column 1) and predicted epitopes by EpiScan (column 2) and PInet (column 3) in red on the RBD surface. Probability of prediction by EpiScan and PInet are presented in columns 4 and 5, with F1_score (left) and AUROC (right) values indicated below each prediction. **b** Boxplot comparisons of EpiScan and PInet performance on evaluation metrics, including Precision, Recall, F1_score, AUROC, and AUPR, for DB1 and DB2 datasets. Lines represent confidence intervals, and *p*-values were calculated using a Wilcoxon test.

This study presented a comprehensive evaluation and analysis of the performance and generalization capabilities of EpiScan, a self-attention-based convolutional neural network model, compared with PInet, a graph neural network-based model. The comparison was based on epitope prediction results for five representative antigen–antibody complexes (Fig. 2a) and performance metrics across two distinct datasets (Fig. 2b). For each BCE type, the representative antigen structure was utilized, as shown in Supplementary Table 2. In Fig. 2a, every amino acid residue on the antigen surface is assigned an epitope probability score, indicating its potential as a component of the antibody binding site (epitope). For EpiScan, this score is derived from the predictive model’s output, a probability matrix of dimensions (Ag-seq-length, 2), where the softmax function assists in extracting the probability of each antigen residue being part of the epitope, we utilize the last dimension of this matrix to articulate the epitope probability.

Figure 2a demonstrates that EpiScan and PInet exhibited varying degrees of success in epitope prediction across the five complexes. The $F1{\_score}$ and ${AU}{ROC}$ highlighted the strengths and limitations of each method, emphasizing the importance of selecting the appropriate approach for epitope prediction tasks. In several cases, EpiScan outperformed PInet, largely due to its ability to consider the interrelationships between V_H_ and V_L_, including the interactions between the CDR_S_ and FRs. EpiScan individually models the interactions between the antigen and V_H_ or V_L_ of the antibody, allowing it to determine the different levels of importance of each chain during binding. On the contrary, PInet does not have this feature. The prediction accuracy of the model varies for different epitope regions. For 3ZKM located in the “helix-loop-helix” motif and 1TZH located in the “sheet-loop-sheet” motif, the prediction accuracy of the model is relatively higher than that of 1NFD located in the loop motif. Moreover, the prediction accuracy of the model for 7ZF8 located in the RBM region is higher than that for 7WRL located in the non-RBM region. In addition to conformational differences, the possible reasons for this difference include the long-tail problem caused by the difference in the number of samples in the target epitope-enriched and non-enriched regions, exacerbating the model’s positive prediction bias towards the enriched region due to data imbalance. Figure 2b illustrates the performance of EpiScan and PInet on DB1, a public database, and DB2, an independent database of SARS-CoV-2 neutralizing antibody–antigen complexes. EpiScan outperformed PInet on all evaluation metrics in DB1, with no significant group differences observed (*p* > 0.05). In DB2, EpiScan showed significant group differences in ${Precision}$, $F1{\_score}$, and ${AUROC}$ (*p* < 0.05), whereas no significant differences were detected for ${Recall}$ and ${AUPR}$.

As an enhancement to our methodological approach, we have included macro-average ROC curves in Supplementary Fig. 3, providing additional insights into the performance of epitope prediction across two datasets. In DB1, a robust evaluation through 5-fold cross-validation exhibits consistent performance with ${AUROC}$ values ranging from 0.70 to 0.72, as evidenced by the closely aligned ROC curves. The nearness of these curves to the top left corner of the plot underscores the effectiveness of our model in distinguishing between the positive class (epitopes) and the negative class (non-epitopes), compared to a random guess which would follow the diagonal dashed line. For DB2, the comparison among various prediction models is illustrated. EpiScan, performs exceptionally well on DB2 with an ${AUROC}$ value of 0.68. In contrast, DeepBindPPI exhibits poorer performance, achieving an ${AUROC}$ of 0.52. The PInet model has an ${AUROC}$ of 0.55, while AbAdapt achieves 0.52. Lastly, the EPI-CNN-GCN model performs least effectively on DB2, with an ${AUROC}$ of 0.51.

We also have adopted a new standard for dividing the training and testing sets, ensuring that the CDR identity among antibodies is less than 70%. This adjustment aims to mitigate the learning benefits derived from antibody homology. The revised dataset, named DB1-cdr70, includes 86 complexes for training and 30 for testing, as detailed in Supplementary Table 6. The performance of EpiScan, PInet, and DeepBindPPI on DB1-cdr70 is summarized in Supplementary Tables 7 and 8. PInet and DeepBindPPI rank closely behind EpiScan, showcasing competitive performance as the subsequent top deep learning models in benchmark DB1 and DB2.

From the results in Supplementary Table 7 and Supplementary Table 8, it is evident that when the training and testing sets are divided based on the criterion of less than 70% CDR sequence identity, all models show a decline in performance compared to the original DB1 test results. Despite the reduced training set (from 132 to 86 samples), EpiScan continues to perform best, validating the robustness of our approach.

Notably, we tuned the parameter γ in the ECA module, which is responsible for the interaction between the antibody CDR and the antigen (see Methods details section). We reduced γ from 2 to 1 (where γ = 2 yielded the best performance in the DB1 test) to increase the convolutional kernel size in the ECA module. We found that increasing the convolutional kernel size improved EpiScan’s performance on the DB1-cdr70 dataset (also performs steadily on the DB2). This improvement is likely because, in datasets with significant CDR differences, focusing too much on the detailed features of the CDR region (with a smaller convolutional kernel) can lead to overfitting. In contrast, a larger convolutional kernel captures global features better, enhancing generalization.

### Evaluation of the performance of EpiScan on DB3 dataset

On another dataset, DB3 (sourced from SEPPA-mAb (2023)^32^), we re-trained and tested the performance of EpiScan. Similar to SEPPA-mAb, we used 860 complexes deposited before July 2017 as the internal training dataset and data deposited after 2017 for testing. We utilized four test sets: DB3-test-193, an independent test set from DB3 comprising 193 antigen-antibody complexes; DB3-test-HIV-36, consisting of 36 HIV Env glycoprotein complexes also sourced from DB3; DB3-test-CoV2-31, containing 31 CoV2 complexes from DB3 (before 2022); and DB2-test-CoV2-24, consisting of 24 CoV2 complexes from DB2 (after 2022). The test results are presented in Table 3.

**Table 3:** Comparison of EpiScan with the SOTA structure-based method SEPPA on **DB3**

Methods	DB3-test-193			DB3-test-HIV-36			DB3-test-CoV2-31			DB2-test-CoV2-24		
	*AUROC*↑	*ACC*↑	*FPR*↓	*AUROC*↑	*ACC*↑	*FPR*↓	*AUROC*↑	*ACC*↑	*FPR*↓	*AUROC*↑	*ACC*↑	*FPR*↓
SEPPA 3.0	0.730	0.776	0.206	0.757	<0.918	>0.058	0.672	0.702	0.252	0.593	0.633	0.314
SEPPA-patch	0.774	0.790	0.196	0.835	<0.918	>0.058	0.657	0.655	0.304	**0.694**	0.714	0.240
SEPPA-mAb	**-**	**0.873**	**0.097**	-	0.918	**0.058**	-	0.753	0.224	-	0.738	0.202
**EpiScan**	**0.864**	0.811	0.150	**0.906**	**0.919**	0.067	**0.778**	**0.780**	**0.182**	0.684	**0.821**	**0.116**

Bold values indicate the best performance for each metric across methods.

Table 3 provides a detailed comparison of EpiScan’s capabilities relative to the SOTA structure-based method SEPPA, utilizing the DB3 dataset. Four distinct test sets form the basis of this analysis: DB3-test-193, DB3-test-HIV-36, DB3-test-CoV2-31, and DB2-test-CoV2-24.

For the DB3-test-193 dataset, EpiScan’s *AUROC* of 0.864 significantly exceeds that of SEPPA-patch by 0.090. While EpiScan’s *FPR* of 0.150 is impressively lower than SEPPA 3.0’s 0.206, it is slightly higher than SEPPA-mAb’s remarkable *FPR* of 0.097, highlighting SEPPA-mAb’s strength in minimizing false positives in this context. In the DB3-test-HIV-36 set, EpiScan presents an *AUROC* of 0.906, outperforming SEPPA-patch by 0.071. Although EpiScan’s *FPR* of 0.067 is exceptionally low, it is marginally higher than SEPPA-mAb’s *FPR* of 0.058. This slight difference underscores SEPPA-mAb’s efficiency in reducing false positives, a testament to its predictive precision. Examining the DB3-test-CoV2-31 dataset, EpiScan’s *AUROC* of 0.778 is substantially higher than SEPPA 3.0’s 0.672. However, its *FPR* of 0.182, while lower than SEPPA-patch’s 0.304, is higher than SEPPA-mAb’s 0.224. Within the DB2-test-CoV2-24 set, EpiScan’s *AUROC* of 0.684 demonstrates competitive performance. Despite its accuracy, EpiScan’s *FPR* of 0.116, though significantly better than SEPPA 3.0’s 0.314, is not as optimized as SEPPA-mAb’s *FPR* of 0.202. This comparison reflects SEPPA-mAb’s capability to maintain a lower false positive rate, emphasizing its precision in this dataset.

Overall, the data indicate that EpiScan exhibits superior performance in terms of *AUROC* and *ACC* across all test sets, with consistently higher *AUROC* compared to the SEPPA methods. The results underscore EpiScan’s effectiveness and reliability as a predictive tool in antigen-antibody interaction studies.

### Evaluation of the performance of EpiScan on DMS-3104 dataset

We derived the DMS-3104 dataset from the studies^33,34^ which includes 3104 anti-RBD antibodies and their corresponding 12 targeted hot regions on the antigen, identified through deep mutational scanning (DMS). Given that DMS does not provide direct and explicit antigen-antibody interaction sites due to the lack of crystal structure resolution, we adapted the evaluation of EpiScan on DMS data into a targeted epitope classification problem. Specifically, we merged the 12 DMS escape regions into four epitope classes (class1-class4), ensuring minimal overlap between the classes, as illustrated in Supplementary Fig. 5.

When the EpiScan model outputs predictions for antibody-specific epitope sites, we assign the predicted target regions to one of four classes based on the following rule: the predicted epitope site is allocated to the class with which it has the largest intersection (i.e., the class for which the intersection of the model output and the class sites is the greatest). Mathematically, this can be expressed as:

Let *P* be the predicted epitope site, and *C1, C2, C3, C4* be the four classes. The predicted class *Cp* is given by:

$${C}_{p}={{{\backslash }}{argmax}}_{{C}_{i}}({{|}}P\cap {C}_{i}{{|}})\,{for\,i}=1,2,3$$

where ${|P}\cap {C}_{i}|$ denotes the size of the intersection between the predicted site *P* and the class sites ${C}_{i}$.

By applying this rule, we have established a method to evaluate the specificity of epitope predictions using DMS data. We have utilized three different datasets as training sets to assess the impact of varying data samples on the model’s antibody sensitivity, with the results presented in Table 4.

**Table 4:** The table presents the performance metrics of the EpiScan model re-trained on different datasets and tested on the **DMS-3104** dataset

Training Dataset	*Precision*↑	*Recall*↑	*AUROC*↑	*AUPR*↑	*F1-score*↑
DB1	0.417	0.434	0.618	0.289	0.374
DB3*	0.539	0.536	0.712	0.412	0.483
DB3 + DB2	0.576	0.588	0.788	0.568	0.554

The training datasets include **DB1,** DB3* (**DB3** excluding the **DB3-test-CoV2-31** dataset), and a combined dataset of **DB3** and **DB2**. The metrics reported are *Precision*, *Recall*, *AUROC*, *AUPR*, and *F1-score*.

**Fig. 3:**
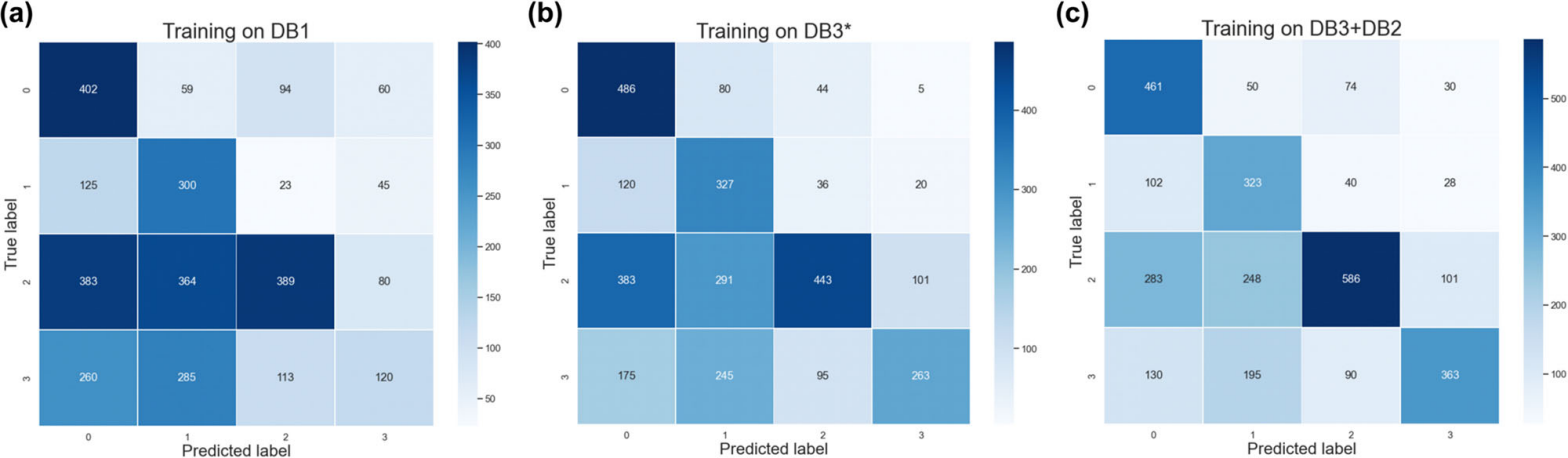
Confusion matrices depicting the performance of the models trained on different datasets. **a** DB1, (**b**) DB3*, and (**c**) DB3 + DB2. The matrices illustrate the distribution of true versus predicted labels, providing insight into the classification accuracy and error patterns across the datasets.

EpiScan trained on the **DB1** dataset yielded a *Precision* of 0.417, *Recall* of 0.434, *AUROC* of 0.618, *AUPR* of 0.289, and an *F1*-*score* of 0.374. When trained on the DB3* dataset, the model’s performance improved, achieving a *Precision* of 0.539, *Recall* of 0.536, *AUROC* of 0.712, *AUPR* of 0.412, and an *F1*-*score* of 0.483. The highest performance across all metrics was observed when the model was trained on the combined **DB3** + **DB2** dataset, with a *Precision* of 0.576, *Recall* of 0.588, *AUROC* of 0.788, *AUPR* of 0.568, and an *F1*-*score* of 0.554. These observations indicate that integrating multiple datasets for model training can significantly enhance model performance. The diversity and richness of information available in the combined DB3 + DB2 dataset likely provide a more comprehensive representation of the epitope space, enabling the model to learn more generalized features that are effective across different datasets. Figure 3 has been plotted to further investigate the predilection of the model for predicting CoV2 epitopes.

From Fig. 3, it is observable that when the EpiScan model is trained on datasets excluding CoV2 complexes, the model’s false positives are predominantly concentrated in Class1 and Class2 epitope regions, which are areas of the Receptor Binding Domain (RBD) with the highest exposure level. Training with the DB1 dataset, the model particularly underperforms in predicting Class3 and Class4 epitopes, with more than two-thirds of the epitope predictions falling within Class1 and Class2. When trained with DB3*, the model shows some improvement in prediction accuracy but still exhibits a bias towards Class1 and Class2. This suggests that without training on CoV2 data, the model demonstrates lower sensitivity to CoV2-specific antibodies, favoring predictions towards potential RBD candidate epitopes (i.e., the immunogenically stronger Class 1 and Class 2). However, with the inclusion of a small amount of CoV2 complex samples, the model’s sensitivity towards Class 3 and Class 4 specific antibodies increased, thereby enhancing its ability to predict anti-RBD specific epitopes. This also supports our statement that training a highly accurate and generalizable specificity epitope prediction model using existing complex data alone is challenging. Tuning the model on data specific to particular scenarios is a worthwhile approach, which is a significant reason for deploying models tailored to specific strains on our web-server.

### Performance of model components

As listed in Table 5, the effect of different blocks on the EpiScan model performance was evaluated by modifying the models, such as by removing or keeping specific components, including the Hinge block and the V_H_/V_L_ separation. The results indicated that the original EpiScan model with ECA/Rotation blocks achieved the highest performance in terms of ${Precision}$ (0.239 ± 0.019), ${Recall}$ (0.776 ± 0.038), ${AUROC}$ (0.715 ± 0.008), ${AUPR}$ (0.304 ± 0.009), $F1{\_score}$ (0.338 ± 0.021) and ${MCC}$ (0.275 ± 0.018). This experiment highlighted the crucial role of each component in the EpiScan model’s overall performance. Removing any of these components leads to a drop in performance, emphasizing the importance of considering all these factors when designing and optimizing models for predicting antibody–antigen interactions.

**Table 5:** Impact of different blocks on EpiScan model performance

Methods	ECA/Rotation	*Precision*↑	*Recall*↑	*AUROC*↑	*AUPR*↑	*F1_score*↑	*MCC*↑
**EpiScan**	+/+	0.239 ± 0.019	0.776 ± 0.038	0.715 ± 0.008	0.304 ± 0.009	0.338 ± 0.021	0.275 ± 0.018
	+/−	0.220 ± 0.025	0.767 ± 0.011	0.703 ± 0.005	0.302 ± 0.009	0.322 ± 0.017	0.269 ± 0.017
	−/+	0.238 ± 0.021	0.602 ± 0.012	0.650 ± 0.006	0.240 ± 0.008	0.280 ± 0.016	0.213 ± 0.014
	−/−	0.200 ± 0.020	0.630 ± 0.015	0.645 ± 0.007	0.235 ± 0.010	0.275 ± 0.018	0.205 ± 0.015
**EpiScan**|Hinge	+/+	0.230 ± 0.018	0.750 ± 0.035	0.705 ± 0.007	0.295 ± 0.008	0.325 ± 0.020	0.266 ± 0.011
	+/−	0.210 ± 0.024	0.690 ± 0.010	0.693 ± 0.004	0.272 ± 0.009	0.310 ± 0.016	0.260 ± 0.016
	−/+	**0.298** **±** **0.020**	**0.509** **±** **0.013**	**0.630** **±** **0.005**	**0.210** **±** **0.008**	**0.220** **±** **0.015**	**0.156** **±** **0.011**
	−/−	0.190 ± 0.019	0.525 ± 0.014	0.615 ± 0.006	0.205 ± 0.009	0.187 ± 0.017	0.132 ± 0.012
**EpiScan** | HL	+/+	0.235 ± 0.019	0.665 ± 0.037	0.700 ± 0.008	0.289 ± 0.009	0.303 ± 0.021	0.253 ± 0.012
	+/−	0.205 ± 0.025	0.675 ± 0.011	0.678 ± 0.005	0.259 ± 0.009	0.290 ± 0.017	0.246 ± 0.015
	−/+	0.203 ± 0.021	0.660 ± 0.012	0.665 ± 0.006	0.247 ± 0.008	0.285 ± 0.016	0.240 ± 0.013
	−/−	0.195 ± 0.020	0.541 ± 0.012	0.610 ± 0.007	0.202 ± 0.010	0.185 ± 0.018	0.170 ± 0.014
**EpiScan**|Hinge|HL	+/+	0.225 ± 0.019	0.535 ± 0.036	0.610 ± 0.008	0.210 ± 0.009	0.220 ± 0.021	0.194 ± 0.011
	+/−	0.195 ± 0.025	0.565 ± 0.011	0.603 ± 0.005	0.225 ± 0.009	0.190 ± 0.017	0.191 ± 0.015
	−/+	0.193 ± 0.021	0.455 ± 0.012	0.581 ± 0.006	0.193 ± 0.008	0.140 ± 0.016	0.116 ± 0.012
	−/−	0.185 ± 0.020	0.440 ± 0.015	0.535 ± 0.007	0.198 ± 0.010	0.136 ± 0.018	0.106 ± 0.013

EpiScan models with modified configurations are denoted by specific labels. “ECA/Rotation” column indicates whether the corresponding block is used (+) or not used (−) in the model. “**EpiScan**|Hinge” refers to the model with the Hinge block removed, while “**EpiScan** | HL” represents a model without V_H_ and V_L_ separation, but with direct completion of the antibody and antigen reaction. Additionally, the label “**EpiScan**|Hinge|HL” denotes a model that simultaneously disregards both the Hinge block and V_H_/V_L_ separation. Performance metrics such as Precision, Recall, AUROC, AUPR, F1_score and MCC are reported for each model configuration.

The importance of the Hinge block in the model was evident when it was removed (**EpiScan**|Hinge), as the performance metrics decreased slightly. The FRs and CDRs plays a critical role in maintaining the structural stability and flexibility of the antibody, allowing it to bind to various antigens with high specificity and affinity^35^. The inclusion of Hinge block in the EpiScan model enables the model to better capture antibody binding specificity by the coupling properties between FRs and CDRs, thereby improving the performance of predicting antibody-antigen interactions.

Similarly, the removal of V_H_/V_L_ separation (**EpiScan** | HL) resulted in decreased performance metrics. The V_H_ and V_L_ are essential components of an antibody’s structure, and their correct separation and interaction are crucial for the antibody’s function. Including V_H_ and V_L_ separation in the EpiScan model can enhance the simulation of the complex structural and functional relationships between these chains, leading to improved accuracy of the predictions of antibody–antigen interactions. The lowest performance was observed when the Hinge block, V_H_/V_L_ separation were disregarded simultaneously (**EpiScan**|Hinge|HL), further demonstrating the significance of these components in the model.

The removal of the Rotation module alone has a minimal effect on the overall performance of EpiScan but a more significant effect on the model’s stability (see the next summary for details). In conclusion, the incorporation of the Hinge block and V_H_/V_L_ separation in the EpiScan model is essential for achieving high performance in predicting antibody–antigen interactions. These components provide an accurate representation of the complex structural and functional relationships within antibodies, leading to improved model performance.

**Fig. 4:**
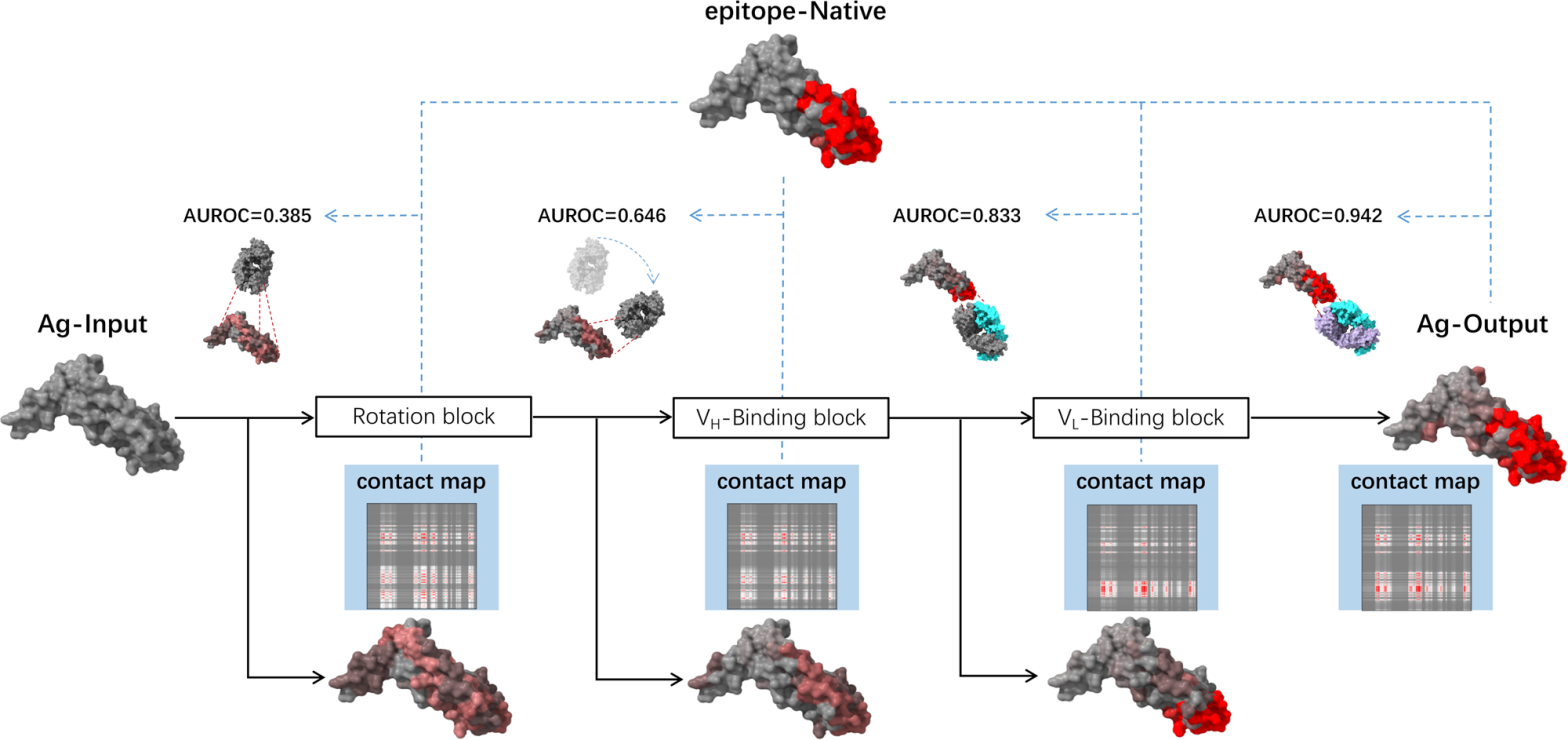
Visualization and analysis of the epitope prediction via EpiScan’s internal blocks (PDB 1TZH). The figure, from left to right, demonstrates the output of the EpiScan model at different stages of epitope prediction, namely the Rotation block, VH-Binding block, and VL-Binding block. The AUROC scores corresponding to each stage are also presented.

Figure 4 offers an illustrative representation of how the EpiScan model operates at varying computational blocks to predict antibody-specific antigen epitopes. The model’s performance at each stage is evaluated using the ${AUROC}$ scores. The first computational block, the Rotation block, is responsible for simulating the translation and rotation of the antibody. Using this mechanism, the model attempts to identify the most probable binding region with the antigen. Before entering the Rotation block, the ${AUROC}$ score is 0.385, indicating poor initial discrimination. After passing through the Rotation block, which simulates antibody movement to identify likely binding regions, the ${AUROC}$ improves to 0.646. The model then proceeds to the next stage, the V_H_-Binding block. This block is responsible for initiating the reaction between the heavy chain (V_H_) of the antibody and the antigen, pinpointing the binding amino acid residues. This process enhances the prediction accuracy, reflected in the improved ${AUROC}$ score of 0.833. Subsequently, the model processes the information through the V_L_-Binding block, which builds upon the heavy chain-antigen recognition. During this stage, the reaction between the light chain (V_L_) of the antibody and the antigen takes place. This further includes possibly overlooked amino acid epitopes, enhancing the model’s comprehensive epitope prediction capability, the ${AUROC}$ reaches 0.942, though with some false positives. Overall, the stage-wise ${AUROC}$ scores demonstrate EpiScan’s capability in incrementally improving prediction performance through coordinated blocking representing key aspects of antibody-antigen binding. The analysis also reveals opportunities to enhance early positioning discrimination and reduce late-stage false positives.

### Robustness evaluation

**Fig. 5:**
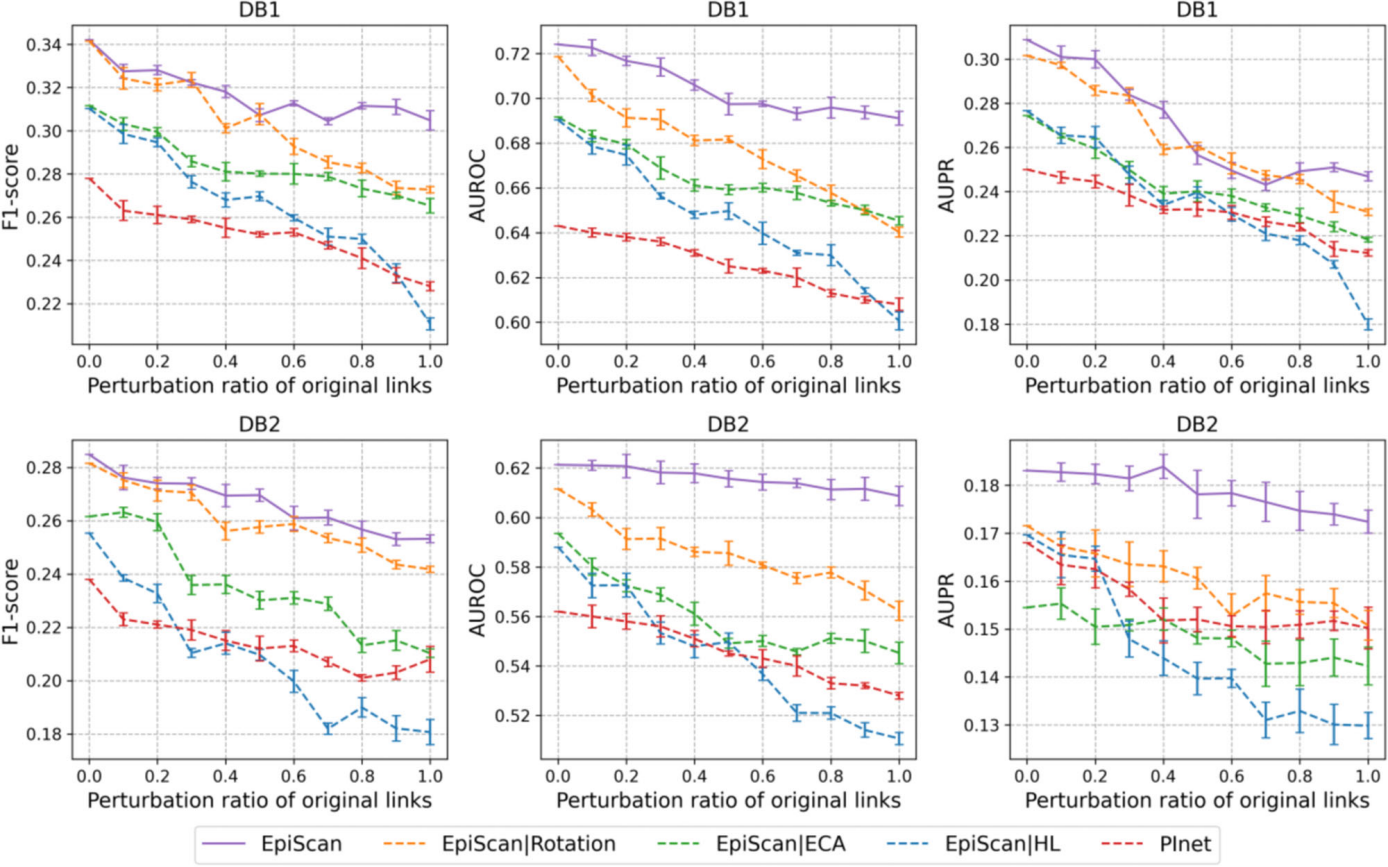
Robustness evaluation of EpiScan and PInet on DB1 and DB2 datasets. The figure displays F1_score, AUROC, and AUPR of baseline predictions against link perturbations with varied ratios of random additions or removals. The label “EpiScan | Rotation” denotes the EpiScan model with the Rotation block removed. Similarly, “EpiScan | ECA” refers to the model with the ECA block removed, and “**EpiScan** | HL” represents a model without V_H_ and V_L_ separation, but with direct completion of antibody and antigen reaction.

Robustness was evaluated on DB1 and DB2 datasets by using EpiScan and PInet models, and the results are shown in Fig. 5.

Different resulting datasets were generated by randomly adding or deleting different proportions of original links to evaluate the robustness of the model. Disturbance characteristic curves were then recalculated for $F1{\_score}$, ${AUROC}$, and ${AUPR}$. Figure 5 shows that the performance of the evaluation indicators of “EpiScan|Rotation” and “**EpiScan** | HL” models declined rapidly, indicating sensitivity to changes in datasets. The PInet model’s performance was better than that of the “**EpiScan** | HL” model, particularly on the DB2 dataset, when the disturbance ratio was high (>60%). The geometric topology modeling of the PInet model enabled it to extensively represent the network structure information, thereby providing some adaptability to high-scale disturbances. Meanwhile, the “**EpiScan**|Rotation” model was observed to be sensitive to interference. By comparison, **EpiScan** proved to be more stable, particularly in terms of $F1{\_score}$ and ${AUROC}$ indices. The Rotation block that estimates the coordinate changes demonstrated some similarity to molecular docking, and it improved the anti-jamming ability of the model. The “**EpiScan**|Rotation” model has a flexible simulation that explains why it is sensitive to interference. The V_H_/V_L_ separation calculation mechanism used to simulate the binding of antibody and antigen contributed to the edge prediction problem under high disturbance. Further, compared with different EpiScan variant models, the separation of Rotation modules with V_H_ and V_L_ enhanced the model’s robustness.

### Effects of different types of input features

In Methods details section, the input features used in EpiScan training based on sequence and structure information were explained in detail. This section focuses on investigating the effects of different types of input features on EpiScan performance in predicting epitopes. The input features are classified into six categories: (i) three-dimensional atomic coordinates of the antigen structure, (ii) solvent accessible surface area, (iii) local amino acid contact maps, (iv) conservative maps containing evolutionary information of antigen sequences, (v) protein language model coding of antibody sequences, and (vi) one-hot encoding and amino acid physicochemical properties coding for the antibody sequence.

**Fig. 6:**
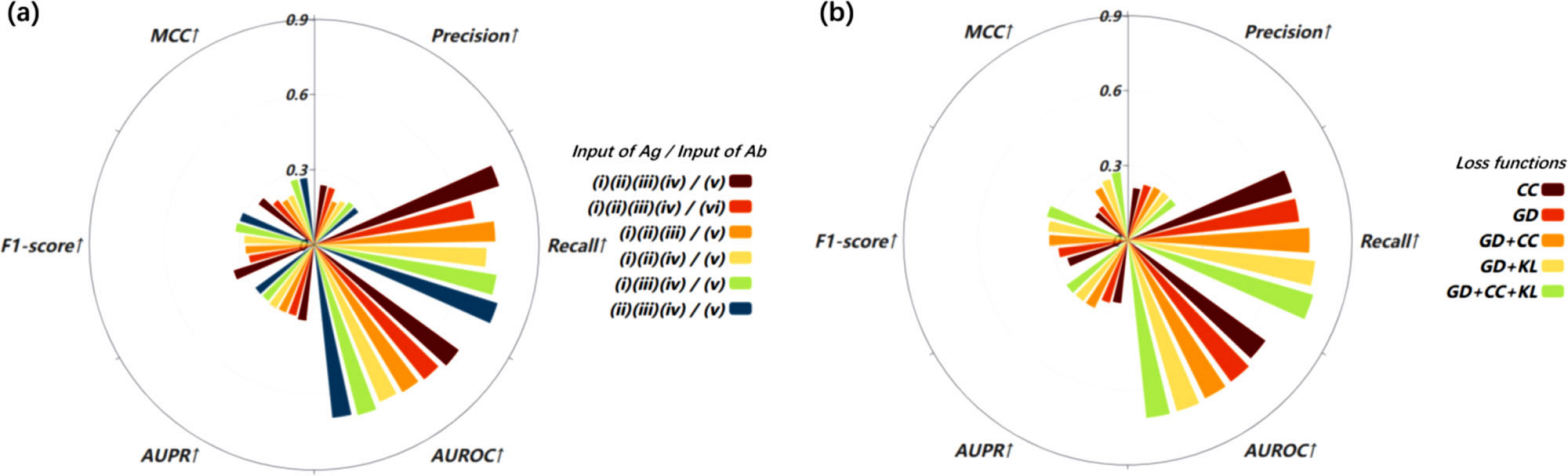
Ablation experiments of the EpiScan model on DB1. **a** Effects of various input feature combinations on EpiScan performance. The input features include (i) Three-dimensional atomic coordinates of the antigen structure, (ii) Solvent accessible surface area, (iii) Local amino acid contact maps, (iv) Conservative maps with evolutionary information of antigen sequences, (v) Protein language model coding of antibody sequences, and (vi) One-hot encoding and amino acid physicochemical properties coding for antibody sequence. **b** Effects of the combination of different loss functions on the performance of EpiScan.

Supplementary Table 3 demonstrates that the combination of all four antigen features (i–iv) with the protein language model coding of antibody sequences (v) achieved the highest performance in terms of ${Precision}$, ${AUPR}$, $F1{\_score}$ and ${AUROC}$. This finding highlighted the effectiveness of incorporating the structural and evolutionary information of the antigen, in addition to the protein language model representation of the antibody, to achieve enhanced accuracy in predicting epitopes. Furthermore, protein language model coding (v) outperformed one-hot encoding and physicochemical property coding (vi) for the antibody sequence in terms of ${Recall}$, ${AUROC}$, ${AUPR}$, and $F1{\_score}$. This finding indicated that the language model encoding representation can better capture the complex relationships between antibody sequences and their binding epitopes. Importantly, omitting any one of the antigen features (i–iv) led to a decline in performance, illustrating the significance of considering all these features in predicting accurate epitopes. Overall, this study emphasized the crucial role of input features selection in EpiScan performance and advocated for a combination of structural, evolutionary, and sequence-based representations to improve epitope prediction accuracy. The presentation in Fig. 6a offers a more intuitive visualization.

### Effects of loss functions on EpiScan performance

The EpiScan model, which is developed for predicting antigen–antibody binding epitopes, faces the challenge of data imbalance due to the relatively small number of antigen epitopes than non-epitope data points. Thus, the effects of different loss functions on EpiScan performance was investigated to address this issue. Supplementary Table 4 presents the effects of various combinations of loss functions on the performance of EpiScan. The loss functions considered include cross correlation (CC) loss, generalized dice (GD) loss, and Kullback–Leibler (KL) divergence.

The results demonstrated that the best performance was achieved by combining all three loss functions. This combination effectively addressed the data imbalance issue and improved the overall performance of EpiScan in predicting antigen–antibody binding epitopes. Furthermore, using CC loss alone led to a significant increase in EpiScan’s performance across all evaluation metrics compared with using GD loss alone. Combining GD loss with either KL divergence or CC loss (i.e., GD + KL or GD + CC) resulted in a slightly enhanced performance. The results underscored the importance of selecting suitable loss functions for tackling the data imbalance challenge in the training process of EpiScan. The presentation in Fig. 6b offers a more intuitive visualization.

### Quantitative mapping of high-throughput neutralizing antibodies on the SARS-CoV-2 RBD reveals variable epitope immunogenicity

**Fig. 7:**
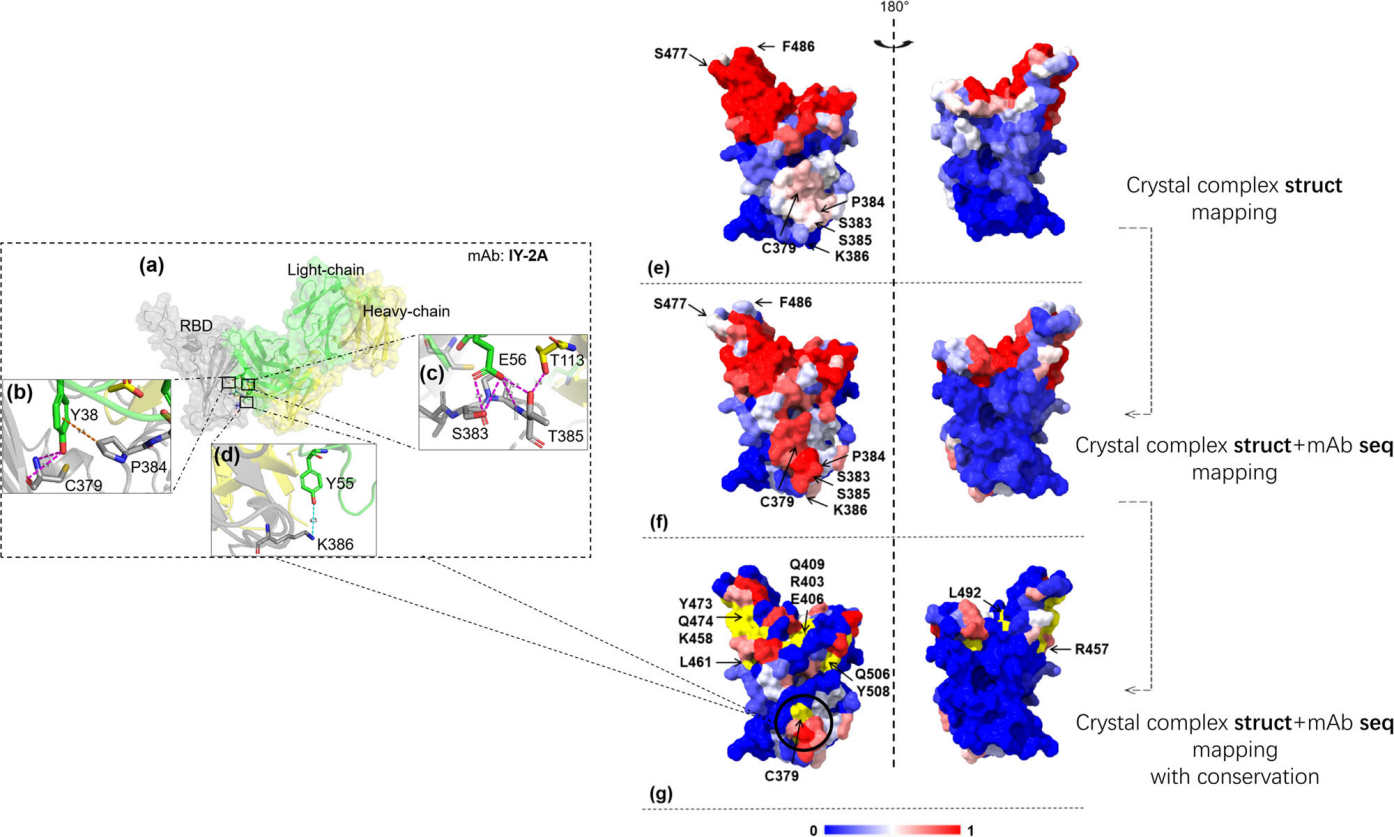
Distribution of antibody-RBD interfaces for neutralizing antibodies of SARS-CoV-2 (PDB:6XC4 chain A). Different colors indicate the prevalence of group-specific sites on the antigen interface, with red representing higher prevalence and blue representing lower prevalence. **e** Mapping of specific epitopes from existing antigen-antibody complexes. **f** EpiScan model-based mapping of specific epitopes on the antigen protein using high-throughput BCR antibody sequencing data. **g** Comprehensive map of specific sites based on epitope conservation, immunogenicity, and immune escape scores. The closer to red, the higher the immunogenicity and conservation, while the closer to blue, the lower the immunogenicity/conservation. Yellow sites warrant particular attention and are suitable for use as vaccine epitopes. Conformational analysis targeting the hot region (379, 383-386) of the IY-2A is conducted. **a** The RBD is shown in gray; the light chain and heavy chain of IY-2A are shown in green and yellow, respectively. **b** Two H-bonds between Y38 and C379. And the hydrophobic interaction between the Y38 and P384. **c** The hydrogen interaction of E56-S383, E56-T385 and T113-T385. **d** The electrostatic interaction between Y55 and K386.

The distribution of the antibody–receptor binding domain (RBD) interface reflects the prevalence of group-specific sites on the antigen interface of different neutralizing antibodies of SARS-CoV-2 (wild-type, WT), as depicted in Fig. 7.

The map in Fig. 7e, which was based on the specific epitopes of existing antigen–antibody complexes^36^, illustrated different colors representing varying levels of prevalence, with red indicating a high epidemic frequency and blue indicating low popularity. Furthermore, the specific epitopes on the antigen protein were mapped using the EpiScan model, which utilized high-throughput BCR antibody sequencing data, as shown in Fig. 7f. The higher frequency of neutralizing antibody binding for S477 and F486 in Fig. 7e is evident in Fig. 7f, likely due to immune escape by mutations at this site. Indeed, in the later stages of the pandemic, it was observed that neutralizing antibodies exhibited a higher affinity towards site regions other than the S477 and F486 sites^33,37^ Meanwhile, specific epitope mapping using high-throughput BCR sequencing data, contrary to the complex statistics, the K386 locus. Previous studies have shown that site 386 is one of the representative targeting sites of RBD neutralizing antibody^37^. Figure 7g presents a comprehensive evaluation of epitope conservation, immunogenicity, and immune escape score, compiling a map of specific sites. The yellow site was identified as highly important and could serve as a valuable vaccine epitope. The red and yellow hotspots in Fig. 7g partially coincide with related studies^38^. Specifically, the sites Q409, E406, R403, Y473, Q474, K458, R457, L492, Q506, Y508, L461, and C379 were marked as potential vaccine epitopes. These sites were selected based on high sequence conservation and high functional conservation^39^, combined with low immune escape score^40^ and high immunogenicity score calculated by EpiScan. It is noteworthy that recent studies have demonstrated a significant overlap between the epitope regions targeted by broad-spectrum neutralizing antibodies in non-RBM areas and the amino acid positions C379, S383-K386 predicted by EpiScan^41^. We have analyzed the potential reasons for the high antibody affinity associated with non-RBM areas(C379, S383-K386). The non-bonded interactions play the vital roles for antibody-antigen complex binding dynamics. Based on the analysis of the IY-2A(IY-2A interacts with a conserved conformational epitope and effectively neutralizes diverse sarbecoviruses while accommodating antigenic variations)^41^ and SARS-CoV-2(WT)-RBD interactions, there were a potential network of hydrogen bonds with the RBD through the CDRs of antibody. As shown in Fig. 7, the side chain of Y38 in the heavy chain formed two hydrogen bonds with C379(Y38: OG-C379: NH1, Y38: N-C379: OH). The E56 residue of the light chain and the S383 residue of the RBD were close to each other and engaged in two hydrogen bonds(E56:OE1-S383:HG, E56:HE2-S383: OG). Meantime, another two hydrogen bonds were formed between side chain of E56 and T385 in RBD(E56:OE2-T385:H, E56:OE2-T385:HG1). In addition, T113 in the heavy chain made a potential hydrogen bond with the oxygen atom of T385 in the RBD(T113:HG1-T385:OG1). The bond lengths of the two hydrogen bonds between the T385 in RBD and E56 in light chain and between the T385 and T113 in heavy chain were shorter (2.7 Å and 2.8 Å respectively) than that on the others, suggesting an important role of hydrogen-bonding interactions at this position. The binding interactions made the binding affinity between the antibody and antigen significantly stronger. Furthermore, there also existed electrostatic interaction between arene-OH of Y55 and the N-H function of K386. The hydrophobic interaction between the Y38 residue in light chain and P384 residue in RBD could remain. Through structure affinity analysis, we described one of the possible sources of strong immunogenicity of the hot region(C379, S383-K386), which as a vaccine epitope may stimulate the production of more IY-2A-like broad-spectrum neutralizing antibodies.

### An interactive and user-friendly EpiScan web server

**Fig. 8:**
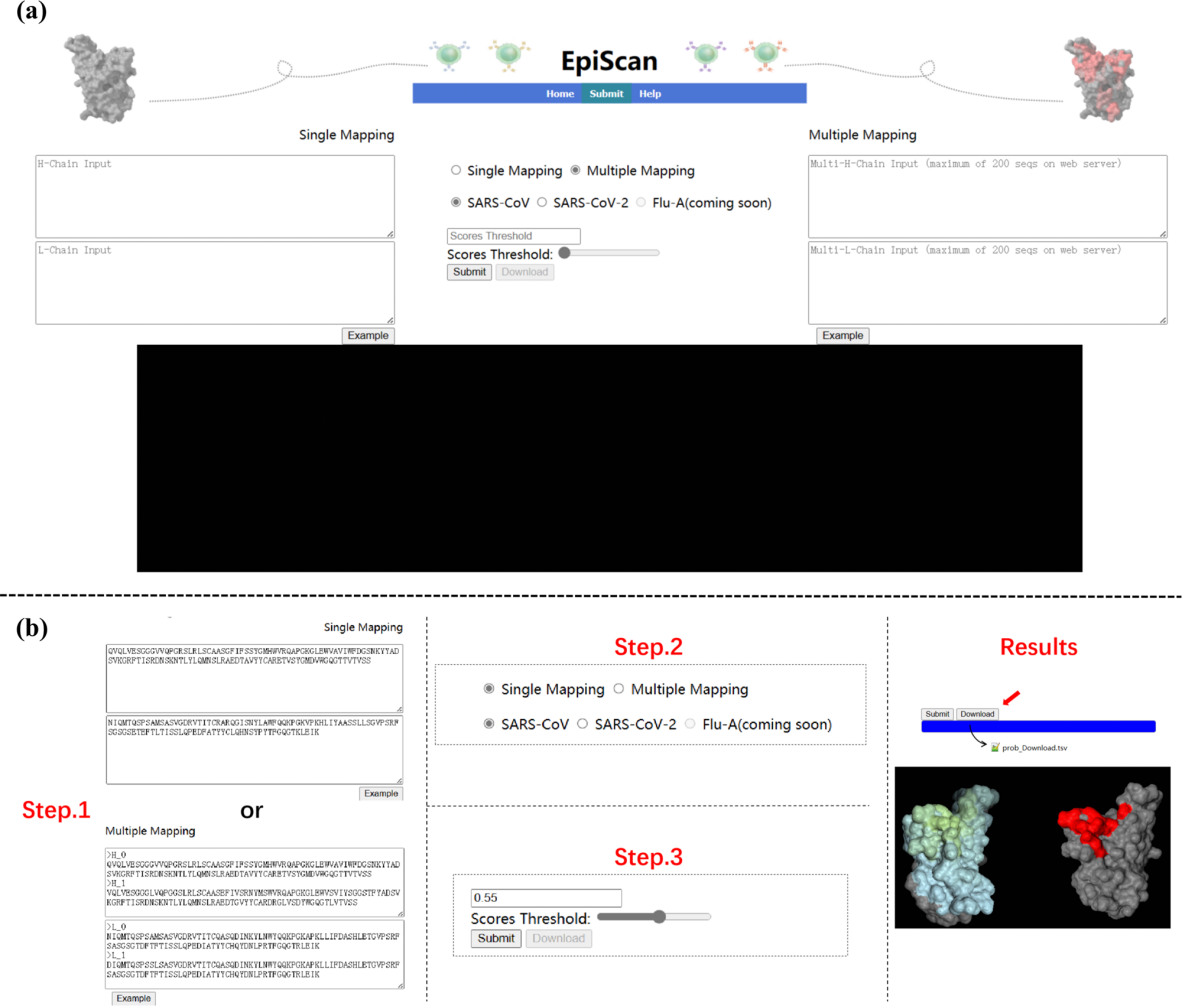
Screenshots to show the submitpage of the EpiScan web server. **a** The page rendering before the task is submitted. **b** The steps to submit (detailed on the help page).

For the convenience of the community, we have developed a user-friendly web server for EpiScan which can be accessed at https://github.com/gzBiomedical/EpiScan, which includes the ‘Home’, ‘Submit’, and ‘Help’ pages (Fig. 8).

Vaccine designers and practitioners can utilize this web server to determine epitope mapping of high-throughput antibody sequences onto specific antigens. We have conducted a comparative analysis of the CALIBER dataset^42^ with DB1, DB2, and DB3, from which we have identified and selected 472 unique antigen-antibody PDB complex samples to serve as an independent test set for the development of our production model on the web-server (Supplementary Table 9). Specifically, after benchmarking the performance of EpiScan through DB1, DB2, and DB3, we constructed a general model using the aforementioned data excluding all samples from these datasets. We then proceeded to test the generalizability of the model on the CALIBER dataset (for which we utilized a subset of 472 samples, selecting 17 humanized antibody samples with CDR identity below 70% compared to the training data; the independent test samples will be selectively adjusted in the future based on practical application scenarios). Based on the actual test results, we set a minimum threshold of 0.80 *AUROC* to determine the optimal general model, EpiScan(general). Subsequently, we fine-tuned EpiScan(general) on 14 external SARS-CoV2 samples from 2023 (Supplementary Table 10) to obtain the specialized model, EpiScan(CoV2). Similarly, we fine-tuned the model on 41 Flu-HA samples (Supplementary Table 10) to derive EpiScan(Flu-A), which is also deployed on our web-server. It’s worth noting that these models were required to achieve an *AUROC* of at least 0.85 on their respective fine-tuning datasets, reflecting a high level of precision and reliability, before being selected for deployment on the cloud server. We are considering, for future work, to incorporate experimental DMS data along with crystallographic data for the fine-tuning and optimization of specific models. We are considering, for future work, to incorporate experimental DMS data along with crystallographic data for the fine-tuning and optimization of specific models. The web server will be continuously updated with specific models for coronaviruses (SARS-CoV, SARS-CoV-2, MERS-CoV) and Influenza A viruses across various strain (we believe the current data is insufficient to train highly accurate general models). EpiScan takes antibody heavy chain and antibody light chain FASTA sequences as input. Users can select the appropriate prediction model based on the antigen type. The users can set their own threshold values, and the prediction results are displayed interactively in a 3D protein visualization on the webpage. Additionally, the results can be downloaded as a TSV file. In the future, researchers will also be able to use this web server to validate the immunogenicity of engineered proteins and infer epitope drift of mutant viral strains. As illustrated in Fig. 7b, Step 3, we have established three reference threshold levels according to [Eq. 19], which are further detailed in Supplementary Fig. 4. Based on the response curve for each antigen-specific model, these reference values indicate thresholds reflecting user preferences for either precision, recall, or a balanced approach between the two. For instance, with SARS-CoV-2, users seeking high precision with minimal false positives may set the threshold near 0.90; those prioritizing a higher recall rate to cover more potential epitopes may adjust the threshold closer to 0.20; and for a more balanced outcome, a threshold around 0.45 would be recommended. The graphical representation offers an optimal threshold to accommodate varying tolerance levels for false positives and false negatives. Beyond the three provided references, users can select an appropriate beta curve based on their tolerances and identify the peak value on the curve for threshold setting. We hope that the introduction of EpiScan can reduce unnecessary experimental procedures and provide hypotheses and supplements for accelerating vaccine development.

## Discussion

This work can be considered as a milestone toward high-throughput specific epitope mapping using only antibody sequences with a determined antigen structure. The modular architecture of EpiScan independently simulates the V_H_ and V_L_ of antibodies, as well as CDRs and FRs, allowing the model to capture the unique roles of different antibody regions in their interaction with antigens. The experimental results demonstrated that EpiScan has good prediction performance on independent datasets of different and similar antigens. The Binding block and Hinge block are important sources that improve the prediction accuracy and generalization characteristics of the model. Furthermore, the design of the V_H_ and V_L_ separation module and the antigen–antibody docking Rotation block, which simulates antibody V_H_ and V_L_ binding with the antigen, significantly contribute to the improvement of the model’s robustness. Ablation experiments demonstrated that the blocks designed in this study and their input features such as structural, evolutionary, and semantic representations are crucial for EpiScan to attain high performance.

In summary, our study presents a robust approach for precise antibody-specific epitope mapping. By leveraging the comprehensive neutralizing antibody sequencing data, we can generate specific antigen epitope maps, which provide valuable resources for identifying high immunogenicity broad-spectrum vaccine epitopes. Notably, through the utilization of EpiScan, we have identified a potential vaccine epitope with remarkable conservation and potent immunogenicity, by mapping the original strain epitopes of SARS-CoV-2 and conducting rigorous conservative analyses. Intriguingly, this promising broad-spectrum epitope has also been independently reported in other investigations, further solidifying its significance and potential application.

However, predicting antibody-specific epitopes is a long-tail problem^43^, as epitope sites account for only a small part of the entire amino-acid sequence. While the effect of sample imbalance was optimized to some extent through the design of the model loss function, the experimental results showed that the false positive rate of the current specific epitope prediction model remains high owing to the influence of the complex physical and chemical properties of the antigen, and multiconformational nature of the protein-protein interactions. Accurately locating the specific epitope is challenging for the model, as any patch on the antigen surface has the potential to become a target for antibodies^14^. Moving forward, EpiScan is poised to incorporate a generative learning module, leveraging a vast repertoire of synthetic data, to substantially heighten the predictive accuracy and generalization capabilities of antibody-specific epitopes. Additionally, by integrating prior knowledge into the model architecture and astutely excluding regions exhibiting suboptimal epitope predictions, a notable reduction in false positives is anticipated.

## Methods and materials

### Methods details

#### Network architecture of feature extraction module

**Fig. 9:**
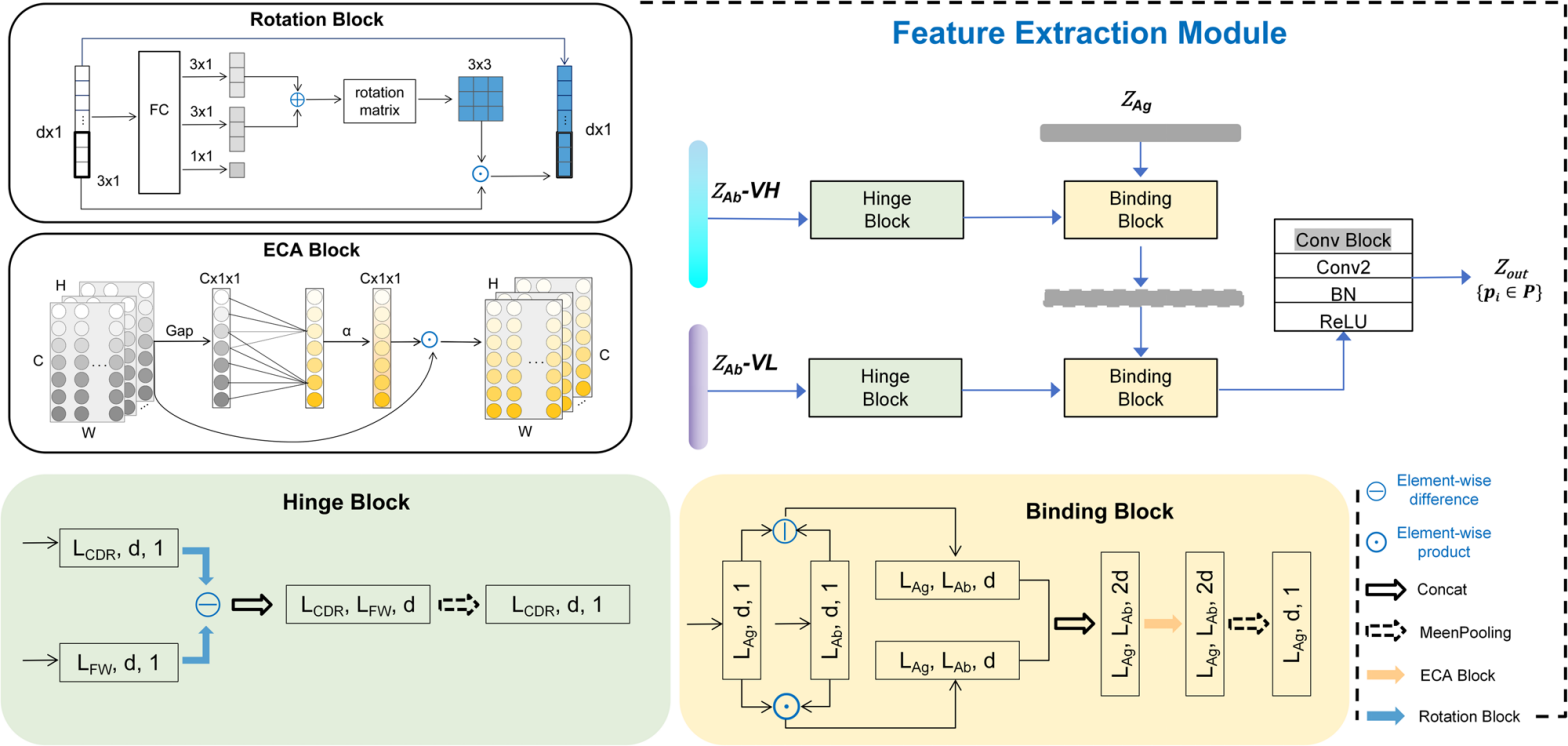
The overall architecture of feature extraction module, comprising four distinct blocks: binding, hinge, rotation, and ECA. The V_H_ progresses through the Hinge block before entering the Binding block simultaneously with the antigen. The output subsequently enters another Binding block with V_L_ via the Hinge block, and proceeds through a convolutional layer to yield the final output.

The Feature extraction module architecture, as shown in Fig. 9, consists of several layers, which applies the ECA module to the input feature maps separately along the antigen and antibody dimensions to weigh the importance of the features.

The first layer in the module is a Rotation block that performs average pooling on the input feature maps along the second dimension, and is followed by a fully connected (Fc) layer that reduces the number of channels to 3 × 1. Next, the efficient channel attention (ECA) module is applied to the input feature maps separately along the antigen and antibody dimensions. The ECA module performs average pooling on the feature maps along the channel dimension and passes them through a 1 × 1 convolution layer with a sigmoid activation function. The weighted feature maps are then passed through a series of three convolutional layers to extract high-level features for downstream tasks, each followed by a batch normalization layer and a rectified linear unit (ReLU) activation function. The convolutional layer has a kernel size of (1,1) × (1,1) with no padding. The details of the configuration of the Feature extraction module are provided in Table 6.

**Table 6:** The configurations of the Feature extraction module

Layer name		Kernel × Stride × Padding	Feature map
Pairs input		—	45 × L_Ag_ 45 × L_Ab_
Rotation	Avg_pool	—	45 × 1
	Fc	—	3 × 1
ECA	Avg_pool	—	45 × L_Ag_ 45 × L_Ab_
	Conv	(3,) × (1,) × (1,)	
	Sigmoid	—	
Conv_1		(1,1) × (1,1) × (0,0)	22 × L_Ag_ × L_Ab_
Batchnorm		—	22 × L_Ag_ × L_Ab_
Activation		—	22 × L_Ag_ × L_Ab_
Conv_2		(1,1) × (1,1) × (0,0)	11 × L_Ag_ × L_Ab_
Batchnorm		—	11 × L_Ag_ × L_Ab_
Activation		—	11 × L_Ag_ × L_Ab_
Conv_3		(7,7) × (1,1) × (3,3)	1 × L_Ag_ × L_Ab_
Batchnorm		—	1 × L_Ag_ × L_Ab_
Activation		—	1 × L_Ag_ × L_Ab_

*Avg_pool* mean pooling, *L*_*Ag*_ length of antigen, *L*_*Ab*_ length of antibody.

#### ECA block

Due to its ability to enhance performance, the incorporation of channel attention in deep learning has garnered significant interest in recent years. One particularly effective approach is the channel attention ECA module proposed by Wang et al., which utilizes a minimal number of additional parameters to achieve a marked improvement in performance^44^. As illustrated in Fig. 9, given the aggregated feature using global average pooling (Gap), ECA module generates channel weights by performing a fast 1D convolution of size $k$ followed by a Sigmoid function ${\rm{\alpha }}$. The kernel size $k$ represents the coverage of local cross-channel interaction, i.e., how many neighbors participate in attention prediction of one channel. Let the output of one convolution block be the $H\times C$ feature map, where $C$ and $H$ are the channel dimension and the length. The ECA module and MaxPooling 2 are stacked together to capture the relationship between adjacent channels and compensate for the loss of information caused by pooling. This helps alleviate the lack of information and improve network performance. Then $k$ is adaptively determined via a function of channel dimension $C$.

1 $$k=\varphi \left(C\right)={\left|\frac{{log }_{2}\left(C\right)+b}{\gamma }\right|}_{{odd}}$$

where ${\left|t\right|}_{{odd}}$ indicates the nearest odd number of $t$. In the present study, we set $\gamma$ and $b$ to be 2 and 1, respectively. Clearly, the mapping function $\varphi$ makes larger size of channels have long-range interaction and vice versa.

#### Hinge block

The Hinge block function is used to couple the antibody frame region with the CDRs, mine the functional coding relationship of the antibody autosequence, and calculate the antibody sequence representation after self-attention.

Given the absolute difference between the ${Z}_{{AbCDR}}$ and ${Z}_{{AbFW}}$, denoted as ${Z}_{{Abdif}}$, the Hinge block function is applied to obtain the output feature map ${Z}_{{Abhinge}}$ as follows:

2 $${Z}_{{Abhinge}}={ECAblock}({Z}_{{Abdif}})$$

Next, the mean pooling operation is applied to ${Z}_{{Abhinge}}$ along the third dimension to obtain the final output feature map ${Z}_{{Ab}}$ as follows:

3 $${Z}_{{Ab}}={meanpooling}({Z}_{{Abhinge}},3)$$

where ${meanpooling}$ is the function that performs the mean pooling operation on ${Z}_{{Abhinge}}$ along the third dimension.

In summary, the Hinge block function is used to couple the FRs with the CDRs, and the output feature map is obtained by applying the self-attention operation followed by mean pooling along the third dimension.

#### Binding block

The Binding block is used to improve the performance of a neural network model by taking into account the relationship between an antibody and an antigen.

Firstly, calculate the absolute difference between the antigen tensor ${Z}_{{Ag}}$ and the antibody tensor ${Z}_{{Ab}}$ by using the following formula:

4 $${Z}_{{dif}}={\rm{|}}{Z}_{{Ag}}\ominus {Z}_{{Ab}}{\rm{|}}$$

Calculate the element-wise multiplication between the input tensor ${Z}_{{Ag}}$ and the reference tensor ${Z}_{{Ab}}$ by using the following formula:

5 $${Z}_{{mul}}={Z}_{{Ag}}\odot {Z}_{{Ab}}$$

Concatenate the tensors ${Z}_{{dif}}$ and ${Z}_{{mul}}$ along the channel dimension by using the following formula and apply the ECA block to the concatenated tensor ${Z}_{{cat}}$:

6 $${Z}_{{cat}}={ECAblock}({concat}({Z}_{{dif}},{Z}_{{mul}},1))$$

Finally, Calculate the mean pooling of the tensor ${Z}_{{cat}}$ along the spatial dimensions by using the following formula:

7 $${Z}_{{mean}}={meanpooling}({Z}_{{cat}},3)$$

By following the above steps and using the formulas provided, the algorithm can efficiently and accurately enhance the features of a neural network model, providing improved results for a variety of tasks.

#### Rotation block

The Rotation block Integrated an algorithm for rotating a set of 3D coordinates around a given axis, designed for use in specific structure feature extraction.

Given a 3D coordinate vector${\boldsymbol{X}}$, a rotation axis vector $a{xis}$, and a rotation angle $r{adian}$, the rotated coordinate vector ${{\boldsymbol{X}}}_{{\boldsymbol{r}}}$ can be calculated using the following formula:

8 $${{\boldsymbol{X}}}_{{\boldsymbol{r}}}= {matri}{x}\_{\exp \left({\rm{cross}}\left({\rm{eye}}\left(3\right),{axi}{s}_{{end}}\right)\cdot {radian}\right)}\,\cdot\, {\boldsymbol{X}}$$

Where ${cross}$ represents the cross product of two matrices, ${eye}(3)$ represents the 3×3 identity matrix, ${matrix\_}\exp$ represents the matrix exponential function, and ${axi}{s}_{{end}}$ is calculated as follows:

9 $${axi}{s}_{{end}}=\frac{{axi}{s}_{{temp}}}{\parallel {axis}\parallel +\epsilon }$$

Here, $\epsilon$ is a small value used to avoid division by zero.

The Rotation block algorithm can be used to enhance the performance of neural network models in structure processing tasks that require rotation of 3D coordinates. By following the above steps and using the formulas provided, the algorithm can efficiently and accurately rotate a set of 3D coordinates around a given axis, providing improved results for a specific structure feature extraction.

#### Cost and optimizer function

A combination of categorical cross-entropy loss (${CC}$)^45^, generalized dice (${GD}$) loss^46^, and Kullback–Leibler (${KL}$) divergence^47^ is used to optimize our model. The formula is as follows:

10 $${LOSS}=0.90{GD}+0.05{CC}+0.05{KL}$$

To minimize the cost function, we used the Adam optimizer function with an initial learning rate of 0.001 and 0.1 decay per 50 steps. In our model, ${CC}$ loss is used to evaluate the difference between the probability distribution obtained from the current training and the true distribution, while ${KL}$ is used to compare the proximity of the two probability distributions.

11 $${CC}=-\frac{1}{H}\mathop{\sum }\limits_{n=1}^{H}{y}_{n}{\mathrm{ln}}\,{{\mathrm{a}}}_{{\mathrm{n}}}+\left(1-{{\mathrm{y}}}_{{\mathrm{n}}}\right){ln}\left(1-{{\mathrm{a}}}_{{\mathrm{n}}}\right)$$

12 $${KL}=\mathop{\sum }\limits_{n=1}^{H}\left[p\left({x}_{n}\right){log}\,p\left({x}_{n}\right)-p\left({x}_{n}\right){log}\, q\left({x}_{n}\right)\right]$$

where ${\rm{H}}$ is the length of the antigen, ${{\rm{y}}}_{{\rm{n}}}$ is an expected value at the position ${\rm{n}}$, and ${{\rm{a}}}_{{\rm{n}}}$ is an actual value from the output layer at the position $n$. $p({x}_{n})$ and $q({x}_{n})$ are the true category probability value and the true category probability value, respectively.

${CC}$ loss and ${KL}$ divergence cannot solve the problem of model performance degradation caused by data imbalance, whereas ${GD}$ loss can integrate different categories of Dice for both balanced and unbalanced data.

13 $${GD}=1-2\frac{{\sum }_{l=1}^{3}{w}_{l}{\sum }_{n}{r}_{{ln}}{p}_{{ln}}}{{\sum }_{l=1}^{3}{w}_{l}{\sum }_{n}{r}_{{ln}}{+p}_{{ln}}}$$

where $n$ is the index of an output position, ${r}_{{ln}}$ is the true value of category $l$ at the position $n$, ${p}_{{ln}}$ is the corresponding predicted probability value and ${w}_{l}$ denotes the weight for each category.

14 $${w}_{l}=\frac{1}{{\left({\sum }_{n=1}^{H}{r}_{{ln}}\right)}^{2}}$$

#### Database curation

**Fig. 10:**
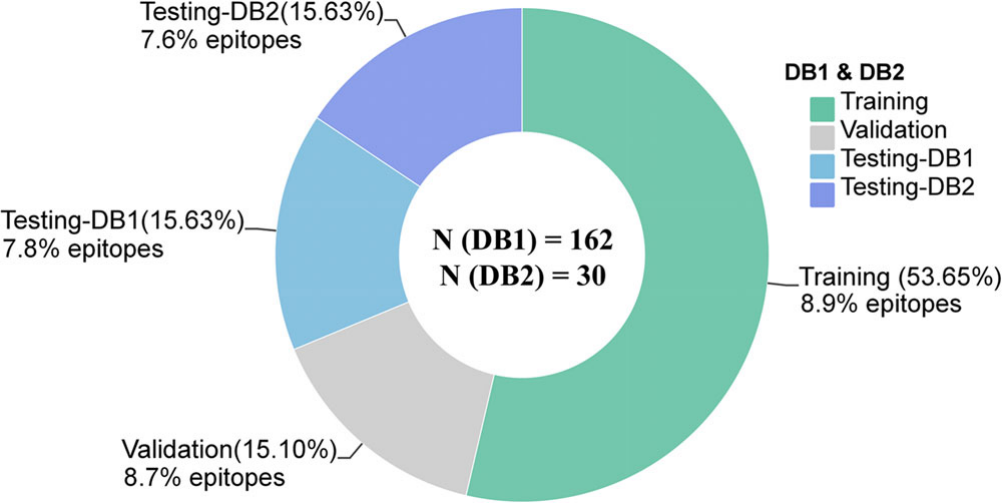
Summary of datasets used for training, validation and testing.

Figure 10 presents a summary of the percentage of residues that form interfaces in the training, validation, and testing sets for the epitope prediction task.

##### The public dataset DB1

The public dataset DB1 was constructed to ensure a fair comparison, adhering to the dataset construction method used by baseline methods. The dataset originates from two main sources: one part is derived from the dataset of EpiPred^16^, which features a unique collection of high-resolution antibody-antigen (Ab-Ag) complex crystal structures, curated from the Structural Antibody Database (SAbDab)^36^. The other part was generated from a separate validation set created from the Docking Benchmark Dataset (DBD) v5^48^. This dataset has been meticulously filtered to ensure structural quality, diversity, and non-redundancy (Supplementary Fig. 1). Serving as a benchmark for antibody-specific epitope prediction, this dataset has been continuously used since 2014. This benchmark dataset comprises 162 Ab-Ag complexes, divided into 103 complexes for training, 29 for validation, and 30 for testing purposes. Furthermore, to mitigate the effects stemming from the homology of antibodies in the training and testing sets, we divided the dataset based on a criterion of CDR identity less than 70%, resulting in the dataset DB1-cdr70 (Supplementary Table 6). On this dataset, we evaluated the most representative deep learning models validated by DB1.

##### The private dataset DB2

To assess the generalizability of Episcan, we assembled DB2, an independent dataset derived from Protein Data Bank (PDB) entries^49^, featuring crystal structures of Coronavirus (SARS-CoV-2, SARS-CoV, and MERS-CoV) antibody-antigen complexes^50^. Redundant complexes were excluded based on the following criteria: resolution superior to 4.5 Å, complete VH and VL, and antigen length of at least 60 amino acids. This filtration process yielded 30 Ab-Ag complexes, which were employed to evaluate the generalization performance of Episcan. (Supplementary Table 1).

##### The public dataset DB3

DB3 originates from the SEPPA-mAb dataset^32^, encompassing 860 antibody-antigen complexes deposited before July 2017 as the internal training dataset. Post-July 2017 data serve as the test set, which includes the independent test set DB3-test-193 with 193 antibody-antigen complexes, the DB3-test-HIV-36 with 36 HIV-related complexes, and the DB3-test-CoV2-31 comprising 31 SARS-CoV-2 complexes deposited before 2022. This dataset has been meticulously curated to ensure structural quality, diversity, and non-redundancy.

##### The private dataset DMS-3104

We derived the DMS-3104 dataset from the studies^32,34^ which includes 3104 anti-RBD antibodies and their corresponding 12 targeted regions on the antigen, identified through deep mutational scanning (DMS). Given that DMS does not provide direct and explicit antigen-antibody interaction sites due to the lack of crystal structure resolution, we adapted the evaluation of EpiScan on DMS data into a targeted epitope classification problem. Specifically, we merged the 12 DMS escape regions into four epitope classes (class1-class4), ensuring minimal overlap between the classes, as illustrated in Supplementary Fig. 5. The DMS-3104 dataset consists of the 4-class epitope region labels, along with the sequences of the antibody light and heavy chains and the corresponding antigen sequences. This dataset represents a significant resource for evaluating epitope targeting and classification methodologies.

#### Input representation of the antigen and antibody structure

In EpiScan’s framework, we associate each amino acid residue of the antigen with a sample point that is represented by a high-dimensional feature vector. This delineation facilitates the comprehensive characterization of antigenic features critical for prediction tasks. Each sample point in the antigenic chain is associated with a 45-dimensional feature vector that encodes essential sequence and structural properties, similar to those utilized by^51^. The feature vector comprises the following components: (i) the 3D coordinates of the amino acids of the antigenic molecules (*d* = 3); (ii) the absolute and relative solvent accessible surface area of the residue, calculated by STRIDE^52^ (*d* = 2); (iii) a local amino acid profile that indicates the frequency of each amino acid type within 8 Å of the residue (*d* = 20); and (iv) a conservation profile for that position across a set of homologous proteins obtained from PSI-BLAST^53^ (*d* = 20).

The antibody amino acid sequence is represented by embeddings from the pre-trained Bepler and Berger^54^ models by connecting the final values of the output layer and all hidden layers (*d* = 45). Their Bi-LSTM neural network model is trained on the protein’s SCOP classification, self-contact map, and sequence alignment of similar proteins. The embeddings capture both local and global features of the protein sequences, distinguishing the embedding method from other approaches that only represent biochemical properties or short-range contexts.

#### Model training and statistical analyses

The model was built in Python using Pytorch as the backend. The workstation used for training the models was implemented on a computer with one CPU running at 2.60 GHz, an NVIDIA GeForce RTX 2080Ti GPU, and 128 GB of memory. The predictions on each antibody–antigen took around 0.02 s to compute. We systematically measured the epitope mapping inference time of EpiScan on a local machine (Intel(R) Xeon(R) Platinum 8255 C CPU @ 2.50 GHz centos 7.6), with the results shown in the Supplementary Table 5.

The judgment criteria generated by different methods were compared with those from the experimental census, yielding methods versus consensus differences. The value of each standard deviation was calculated. The Wilcoxon rank^55^ sum test for the nonparametric test of two populations was applied. The *p*-value matrix was reorganized by pairwise comparisons of the methods.

#### Discordance metrics of local measure

Three performance indices (${Precision}$ [Eq. 15], ${Recall}$ [Eq. 16], $F1{\_score}$ [Eq. 17], ${MCC}$ [Eq. 18], and ${F\_score}$ [Eq. 19]) were calculated using the following equations to evaluate the performance of epitope prediction by a threshold-dependent measurement:

15 $${Precision}=\frac{{TP}}{{TP}+{FP}}$$

16 $${Recall}=\frac{{TP}}{{TP}+{FN}}$$

17 $$F1{\rm{\_}}{score}=\frac{2{TP}}{2{TP}+{FP}+{FN}}$$

18 $${MCC}=\frac{{TP}\times {TN}-{FP}\times {FN}}{\sqrt{\left({TP}+{FP}\right)\left({TP}+{FN}\right)\left({TN}+{FP}\right)\left({TN}+{FN}\right)}}$$

19 $$F{{\_}}{score}=\left(1+{\beta }^{2}\right)\times \frac{{precision}\times {recall}}{\left({\beta }^{2}\times {precision}\right)+{recall}}$$

20 $${ACC}=\frac{{TP}+{TN}}{{TP}+{FP}+{FN}+{TN}}$$

21 $${FPR}=\frac{{FP}}{{FP}+{TN}}$$

where ${TP}$ (true positive) is the number of interacting residues that are correctly predicted as BCEs, ${FP}$ (false positive) is the number of non-interacting residues that are falsely predicted as BCEs, ${TN}$ (true negative) denotes the number of non-interacting sites that are identified correctly, and ${FN}$ (false negative) denotes the number of interacting sites that are identified falsely. ${Precision}$ and ${Recall}$ reflect the prediction tendencies of classifiers. ${Recall}$ indicates the percentage of correct predictions for positive and negative samples. ${Precision}$ shows the percentage of correct positive samples. Where parameter $\beta$ value between 0 and 1 emphasizes ${Precision}$ over ${Recall}$, a $\beta$ value greater than 1 emphasizes ${Recall}$ over ${Precision}$, and a $\beta$ value of 1 equates ${\rm{F\_score}}$ with the $F1{\_score}$.

A trade-off exists between ${Precision}$ and ${Recall}$. In the context of epitope identification, minimizing false positives is critical, especially when pinpointing amino acid hotspots that may lead to immune escape. A higher false positive rate can significantly expand the combinatorial space, complicating immunogen design. Conversely, false negatives (missed detections) can also impact the process by potentially overlooking significant epitope candidates. Supplementary Fig. 2. offers a detailed analysis of both false negative and positive rates observed by EpiScan during the DB2 test. The bar graphs show the Exponential Mean Positive Frequency per Site (%) for different residues within the RBD. Additionally, the protein structure visualization highlights regions with high-frequency false positives (colored green) and false negatives (colored yellow). This comprehensive visual representation underscores the need for improved accuracy and precision in epitope detection methods to enhance immunogen design.

### Discordance metrics of global measure

For global measure, the area under the receiver operating characteristic curve (${AUROC}$) was introduced by Jezewski et al.^56^ and the area under the Precision–Recall curve (${AUPR}$) proposed by Boudet et al.^57^ were used as overall evaluation criteria. ${AUROC}$ (or equivalently c-statistic) is a single measure for evaluating the overall discriminative performance of the ML model. Besides, ${AUROC}$ has been consistently used in prior research dealing with classification. Meanwhile, $A{UPR}$ is considered a robust metric for evaluating models dealing with class imbalances. The value for these metrics ranges from 0 to 1, where a high score indicates good classification capability.

### Reporting summary

Further information on research design is available in the Nature Research Reporting Summary linked to this article.

## Supplementary information

SUPPLEMENTAL INFORMATION

## Data Availability

All original data has been deposited at https://github.com/gzBiomedical/EpiScan.

## Code availability

All code related to the manuscript is available at https://github.com/gzBiomedical/EpiScan, and is publicly available as of the date of publication.

## References

[1] Tsiantoulas, D., Diehl, C. J., Witztum, J. L. & Binder, C. J. B cells and humoral immunity in atherosclerosis. *Circ. Res.* **114**, 1743–1756 (2014). 24855199 10.1161/CIRCRESAHA.113.301145 PMC4066414

[2] Parvizpour, S., Pourseif, M. M., Razmara, J. & Rafi, M. A. Epitope-based vaccine design: a comprehensive overview of bioinformatics approaches. *Drug Discov. Today* **25**, 1034–1042 (2020). 32205198 10.1016/j.drudis.2020.03.006

[3] Bai, X. C., McMullan, G. & Scheres, S. H. W. How cryo-EM is revolutionizing structural biology. *Trends Biochem. Sci.* **40**, 49–57 (2015). 25544475 10.1016/j.tibs.2014.10.005

[4] Hundsberger, H. et al. Assembly and use of high-density recombinant peptide chips for large-scale ligand screening is a practical alternative to synthetic peptide libraries. *BMC Genom.* **18**, 1–10 (2017). 10.1186/s12864-017-3814-3 PMC5463365 28595602

[5] Rawal, K. et al. Identification of vaccine targets in pathogens and design of a vaccine using computational approaches. *Sci. Rep.* **11**, 17626 (2021). 34475453 10.1038/s41598-021-96863-x PMC8413327

[6] Singh, H., Ansari, H. R. & Raghava, G. P. Improved method for linear B-cell epitope prediction using antigen’s primary sequence. *PLoS ONE* **8**, e62216 (2013). 23667458 10.1371/journal.pone.0062216 PMC3646881

[7] Jespersen, M. C., Peters, B., Nielsen, M. & Marcatili, P. BepiPred-2.0: improving sequence-based B-cell epitope prediction using conformational epitopes. *Nucleic Acids Res.* **45**, W24–W29 (2017). 28472356 10.1093/nar/gkx346 PMC5570230

[8] Zhao, L., Wong, L., Lu, L., Hoi, S. C. & Li, J. B-cell epitope prediction through a graph model. *BMC Bioinforma.* **13**, 1–12 (2012). 10.1186/1471-2105-13-S17-S20 PMC3521413 23281855

[9] Minhas, F. U. A. A., Geiss, B. J. & Ben-Hur, A. PAIRpred: partner-specific prediction of interacting residues from sequence and structure. *Proteins* **82**, 1142–1155 (2014). 24243399 10.1002/prot.24479 PMC4329725

[10] Poorinmohammad, N. & Mohabatkar, H. Homology modeling and conformational epitope prediction of envelope protein of Alkhumra haemorrhagic fever virus. *J. Arthropod Borne Dis.* **9**, 116–124 (2015). 26114149 PMC4478412

[11] Porollo, A. & Meller, J. Prediction-based fingerprints of protein-protein interactions. *Proteins* **66**, 630–645 (2007). 17152079 10.1002/prot.21248

[12] Greenbaum, J. A. et al. Towards a consensus on datasets and evaluation metrics for developing B-cell epitope prediction tools. *J. Mol. Recognit.* **20**, 75–82 (2007). 17205610 10.1002/jmr.815

[13] Blythe, M. J. & Flower, D. R. Benchmarking B cell epitope prediction: underperformance of existing methods. *Protein Sci.* **14**, 246–248 (2005). 15576553 10.1110/ps.041059505 PMC2253337

[14] Sela-Culang, I., Ofran, Y. & Peters, B. Antibody specific epitope prediction-emergence of a new paradigm. *Curr. Opin. Virol.* **11**, 98–102 (2015). 25837466 10.1016/j.coviro.2015.03.012 PMC4456244

[15] Hua, C. K. et al. Computationally-driven identification of antibody epitopes. *eLife* **6**, e29023 (2017). 29199956 10.7554/eLife.29023 PMC5739537

[16] Krawczyk, K., Liu, X., Baker, T., Shi, J. & Deane, C. M. Improving B-cell epitope prediction and its application to global antibody-antigen docking. *Bioinformatics* **30**, 2288–2294 (2014). 24753488 10.1093/bioinformatics/btu190 PMC4207425

[17] Pittala, S. & Bailey-Kellogg, C. Learning context-aware structural representations to predict antigen and antibody binding interfaces. *Bioinformatics* **36**, 3996–4003 (2020). 32321157 10.1093/bioinformatics/btaa263 PMC7332568

[18] Gainza, P. et al. Deciphering interaction fingerprints from protein molecular surfaces using geometric deep learning. *Nat. Methods* **17**, 184–192 (2020). 31819266 10.1038/s41592-019-0666-6

[19] Del Vecchio, A., Deac, A., Liò, P. & Veličković, P. Neural message passing for joint paratope-epitope prediction. arXiv preprint arXiv:2106.00757. https://arxiv.org/abs/2106.00757 (2021).

[20] Davila, A. et al. AbAdapt: an adaptive approach to predicting antibody-antigen complex structures from sequence. *Bioinform. Adv.* **2**, vbac015 (2022). 36699363 10.1093/bioadv/vbac015 PMC9710585

[21] Sunny, S., Prakash, P. B., Gopakumar, G. & Jayaraj, P. B. DeepBindPPI: protein-protein binding site prediction using attention based graph convolutional network. *Protein J*. **42**, 276–287 (2023). 10.1007/s10930-023-10121-9 PMC10191823 37198346

[22] Zeng, M., Zhang, F., Wu, F. X., Li, Y. & Wang, J. Protein-protein interaction site prediction through combining local and global features with deep neural networks. *Bioinformatics* **36**, 1114–1120 (2020). 31593229 10.1093/bioinformatics/btz699

[23] Reis, P. B. et al. Antibody-antigen binding interface analysis in the big data era. *Front. Mol. Biosci*. **9**, 945808 (2022). 10.3389/fmolb.2022.945808 PMC9329859 35911958

[24] Fung, K. M., Lai, S. J., Lin, T. L. & Tseng, T. S. Antigen–antibody complex-guided exploration of the hotspots conferring the immune-escaping ability of the SARS-CoV-2 RBD. *Front. Mol. Biosci*. **9**, 797132 (2022). 10.3389/fmolb.2022.797132 PMC8981523 35392535

[25] Saerens, D., Huang, L., Bonroy, K. & Muyldermans, S. Antibody fragments as probe in biosensor development. *Sensors* **8**, 4669–4686 (2008). 27873779 10.3390/s8084669 PMC3705465

[26] Maynard, J. & Georgiou, G. Antibody engineering. *Annu. Rev. Biomed. Eng.* **2**, 339–376 (2000). 11701516 10.1146/annurev.bioeng.2.1.339

[27] Tiller, K. E. & Tessier, P. M. Advances in antibody design. *Annu. Rev. Biomed. Eng.* **17**, 191–216 (2015). 26274600 10.1146/annurev-bioeng-071114-040733 PMC5289076

[28] Candon, M. et al. Advanced multi-input system identification for next generation aircraft loads monitoring using linear regression, neural networks and deep learning. *Mech. Syst. Signal Process.* **171**, 108809 (2022). 10.1016/j.ymssp.2022.108809

[29] Hewage, P., Trovati, M., Pereira, E. & Behera, A. Deep learning-based effective fine-grained weather forecasting model. *Pattern Anal. Appl.* **24**, 343–366 (2021). 10.1007/s10044-020-00898-1

[30] Ge, J., Liang, Y. C., Joung, J. & Sun, S. Deep reinforcement learning for distributed dynamic MISO downlink-beamforming coordination. *IEEE Trans. Commun.* **68**, 6070–6085 (2020). 10.1109/TCOMM.2020.3004524

[31] Diamantaras, K., Vranou, G. & Papadimitriou, T. Multi-input single-output nonlinear blind separation of binary sources. *IEEE Trans. Signal Process.* **61**, 2866–2873 (2013). 10.1109/TSP.2013.2255046

[32] Qiu, T. et al. SEPPA-mAb: spatial epitope prediction of protein antigens for mAbs. *Nucleic Acids Res.* **51**, W528–W534 (2023). 37216611 10.1093/nar/gkad427 PMC10320061

[33] Cao, Y. et al. BA.2.12.1, BA.4 and BA.5 escape antibodies elicited by Omicron infection. *Nature* **608**, 593–602 (2022). 35714668 10.1038/s41586-022-04980-y PMC9385493

[34] Cao, Y. et al. Imprinted SARS-CoV-2 humoral immunity induces convergent Omicron RBD evolution. *Nature* **614**, 521–529 (2023). 36535326 10.1038/s41586-022-05644-7 PMC9931576

[35] Janeway Jr, C. A., Travers, P., Walport, M. & Shlomchik, M. J. The structure of a typical antibody molecule. Immunobiology: *The Immune System in Health and Disease*, *5th edition*, *Garland Science* (2001).

[36] Dunbar, J. et al. SAbDab: the structural antibody database. *Nucleic Acids Res.* **42**, D1140–D1146 (2014). 24214988 10.1093/nar/gkt1043 PMC3965125

[37] Yi, C. et al. Comprehensive mapping of binding hot spots of SARS-CoV-2 RBD-specific neutralizing antibodies for tracking immune escape variants. *Genome Med.* **13**, 1–17 (2021). 34649620 10.1186/s13073-021-00985-w PMC8515915

[38] Cao, Y. et al. Omicron escapes the majority of existing SARS-CoV-2 neutralizing antibodies. *Nature* **602**, 657–663 (2022). 35016194 10.1038/s41586-021-04385-3 PMC8866119

[39] Liu, X. et al. Deep geometric representations for modeling effects of mutations on protein-protein binding affinity. *PLoS Comput. Biol.* **17**, e1009452 (2021). 34347784 10.1371/journal.pcbi.1009284 PMC8366979

[40] Greaney, A. J., Starr, T. N. & Bloom, J. D. An antibody-escape calculator for mutations to the SARS-CoV-2 receptor-binding domain. *Virus evolution* **8**, veac021 (2022). 10.1093/ve/veac021 PMC9092643 35573973

[41] Huang, K. Y. A. et al. Structural basis for a conserved neutralization epitope on the receptor-binding domain of SARS-CoV-2. *Nat. Commun.* **14**, 311 (2023). 36658148 10.1038/s41467-023-35949-8 PMC9852238

[42] Israeli, S. & Louzoun, Y. Single-residue linear and conformational B cell epitopes prediction using random and ESM-2 based projections. *Brief. Bioinforma.* **25**, bbae084 (2024). 10.1093/bib/bbae084 PMC10940830 38487845

[43] Menon, A. K. et al. Long-tail learning via logit adjustment. International Conference on Learning Representations. (2021).

[44] Wang, Q. et al. ECA-Net: efficient channel attention for deep convolutional neural networks. *Proc. IEEE/CVF Conf. Comput. Vis. Pattern Recognit*. pp. 11531–11539, 10.1109/CVPR42600.2020.01155 (2020).

[45] Zhang, Z. & Sabuncu, M. Generalized cross entropy loss for training deep neural networks with noisy labels. *Adv. Neural Inf. Process. Syst*. **31**, 8792–8802 (2018). PMC11747755 39839708

[46] Sudre, C. H., Li, W., Vercauteren, T., Ourselin, S. & Cardoso, M. J. Generalised dice overlap as a deep learning loss function for highly unbalanced segmentations. *Deep Learn Med Image Anal Multimodel Learn Clin Decis Support* **3**, 240–248 (2017). 10.1007/978-3-319-67558-9_28 PMC7610921 34104926

[47] Joyce, J. M. Kullback-leibler divergence. *International Encyclopedia of Statistical Science*. pp 720–722, 10.1007/978-3-642-04898-2_327 (2011).

[48] Vreven, T. et al. Updates to the integrated protein–protein interaction benchmarks: docking benchmark version 5 and affinity benchmark version 2. *J. Mol. Biol.* **427**, 3031–3041 (2015). 26231283 10.1016/j.jmb.2015.07.016 PMC4677049

[49] Berman, H. M. et al. The protein data bank. *Nucleic Acids Res.* **28**, 235–242 (2000). 10592235 10.1093/nar/28.1.235 PMC102472

[50] Raybould, M. I., Kovaltsuk, A., Marks, C. & Deane, C. M. CoV-AbDab: the coronavirus antibody database. *Bioinformatics* **37**, 734–735 (2021). 32805021 10.1093/bioinformatics/btaa739 PMC7558925

[51] Fout, A., Byrd, J., Shariat, B. & Ben-Hur, A. Protein interface prediction using graph convolutional networks. *Adv. Neural Inform. Process. Syst*. **30**, 6533–6542 (2017).

[52] Heinig, M. & Frishman, D. STRIDE: a web server for secondary structure assignment from known atomic coordinates of proteins. *Nucleic Acids Res.* **32**, W500–W502 (2004). 15215436 10.1093/nar/gkh429 PMC441567

[53] Altschul, S. F. et al. Gapped BLAST and PSI-BLAST: a new generation of protein database search programs. *Nucleic Acids Res.* **25**, 3389–3402 (1997). 9254694 10.1093/nar/25.17.3389 PMC146917

[54] Bepler, T. & Berger, B. Learning the protein language: evolution, structure, and function. *Cell Syst.* **12**, 654–669 (2021). 34139171 10.1016/j.cels.2021.05.017 PMC8238390

[55] Lam, F. C. & Longnecker, M. T. A modified Wilcoxon rank sum test for paired data. *Biometrika* **70**, 510–513 (1983). 10.1093/biomet/70.2.510

[56] Narkhede, S. Understanding AUC-ROC Curve: Towards Data Science **26**, 220–227 (2018).

[57] Davis, J. & Goadrich, M. The relationship between Precision-Recall and ROC curves. *Proceedings of the 23rd International Conference on Machine Learning*. pp 233–240 (2006).

[58] Dai, B. & Bailey-Kellogg, C. Protein interaction interface region prediction by geometric deep learning. *Bioinformatics* **37**, 2580–2588 (2021). 33693581 10.1093/bioinformatics/btab154 PMC8428585

