# Supplementary material for: EpiScan: accurate high-throughput mapping of antibody-specific epitopes using sequence information
Source: NPJ Syst Biol Appl. 2024 Sep 9;10:101. doi: 10.1038/s41540-024-00432-7 (PMC11383971; doi:10.1038/s41540-024-00432-7)
Supplement: Supplementary file 1 — SUPPLEMENTAL INFORMATION [file 41540_2024_432_MOESM1_ESM.pdf]

SUPPLEMENTAL INFORMATION

Supplementary Table 1. List of 30 PDB files that constitute the additional dataset(DB2) for epitope prediction

Binds to	PDB ID	Heavy chain	Light chain	Antigen Chain
SARS-CoV2_WT	7X7O	E	F	C
	7XXL	A	C	B
	7Z0X	H	L	R
	7V27	B	A	C
	7VYR	H	L	R
	7WUE	C	D	A
	8DT3	H	L	C
SARS-CoV2_Omicron BA.1	7XCK	A	B	M
	7XCP	H	L	B
	7Y0C	A	B	R
	7Y0V	H	L	R
	7YAD	A	B	M
	7ZF3	H	L	E
	7ZF5	H	L	E
	7ZFA	E	F	B
	7WEE	H	L	E
	7WEF	C	L	E
	7WI0	B	D	A
	7WLC	H	L	E
	7WRL	A	B	R
	7WRZ	H	L	R
	7X1M	A	B	M
SARS-CoV2_Omicron BA.2	7ZF8	H	L	E
	7ZF9	H	L	E
SARS-CoV1	7X7V	H	L	E
	2DD8	H	L	S
MERS-CoV	4XAK	H	L	A
	4ZPV	H	L	R
	4ZS6	H	L	A
	5GMQ	B	C	A

Supplementary Table 2. Five types of BCEs for epitope mapping visualization.

PDB ID	Epitope region(motif)	Heavy chain	Light chain	Antigen Chain
3ZKM	helix-loop-helix	C	D	B
1TZH	sheet-loop-sheet	B	A	W
4G3Y	loop	B	A	C
7ZF8	RBM	H	L	E
7WRL	none-RBM	A	B	R

Supplementary Table 3. Effects of various input feature combinations on EpiScan performance. The input features include (i) Three-dimensional atomic coordinates of the antigen structure, (ii) Solvent accessible surface area, (iii) Local amino acid contact maps, (iv) Conservative maps with evolutionary information of antigen sequences, (v) Protein language model coding of antibody sequences, and (vi) One-hot encoding and amino acid physicochemical properties coding for antibody sequence.

Input of Ag / Input of Ab	*Precision↑*	*Recall↑*	*AUROC↑*	*AUPR↑*	*F1-score↑*
**(i)(ii)(iii)(iv) / (v)**	**0.239±0.019**	**0.776±0.038**	**0.715±0.008**	**0.304±0.009**	**0.338±0.021**
(i)(ii)(iii)(iv) / (vi)	0.236±0.011	0.650±0.015	0.695±0.005	0.292±0.006	0.265±0.020
(i)(ii)(iii) / (v)	0.188±0.015	0.725±0.025	0.687±0.006	0.293±0.007	0.275±0.019
(i)(ii)(iv) / (v)	0.205±0.016	0.690±0.030	0.680±0.007	0.295±0.008	0.280±0.020
(i)(iii)(iv) / (v)	0.221±0.018	0.740±0.035	0.703±0.006	0.290±0.009	0.320±0.022
(ii)(iii)(iv) / (v)	0.219±0.017	0.770±0.022	0.695±0.005	0.291±0.008	0.312±0.018

Supplementary Table 4. Effects of the combination of different loss functions on the performance of EpiScan.

Loss functions	*Precision↑*	*Recall↑*	*AUROC↑*	*AUPR↑*	*F1-score↑*	*MCC↑*
CC	0.210±0.025	0.687±0.011	0.683±0.005	0.252±0.009	0.252±0.017	0.161±0.013
GD	0.232±0.020	0.692±0.011	0.701±0.006	0.260±0.011	0.280±0.012	0.170±0.009
GD+CC	0.238±0.021	0.730±0.011	0.705±0.006	0.301±0.010	0.315±0.018	0.236±0.010
GD+KL	**0.240±0.012**	0.757±0.013	0.713±0.017	0.302±0.009	0.322±0.015	0.255±0.012
**GD+CC+KL**	0.239±0.019	**0.776±0.038**	**0.715±0.008**	**0.304±0.009**	**0.338±0.021**	**0.275±0.018**

Supplementary Table 5. Illustrates the computation time for epitope mapping inference of EpiScan and other baseline deep learning models on a local machine. The table provides detailed timings for feature extraction, prediction, and overall inference.

**Model**	**Wall-clock (per sample)**		
	**Feature Extract**	**Predict**	**Overall inference (local)**
**EpiScan**	**4.12s** (Ab-seq embedding)	**0.05s**	**4.17s**
**PInet**	**-**	**1.17s**	**-**
**DeepBindPPI**	**-**	**0.08s**	**-**
**EPI-CNN-GCN**	**-**	**1.60s**	**-**
**PECAN**	**-**	**1.20s**	**-**

Supplementary Table 6. Training and testing sets of the **DB1** dataset divided according to the criterion of less than 70% CDR similarity among antibodies, denominated as **DB1-cdr70**.

PDB ID in Traning list (86)
['1AHW', '1EO8', '1FJ1', '1FSK', '1H0D', '1I9R', '1IQD', '1JPS', '1KB5', '1KXQ', '1LK3', '1NCA', '1OAZ', '1OB1', '1ORS', '1RJL', '1TQB', '1WEJ', '1YJD', '2ADF', '2AEP', '2ARJ', '2J88', '2JEL', '2Q8B', '2QQN', '2R56', '2UZI', '2VIS', '2VXQ', '2VXS', '2XQB', '2XQY', '2XWT', '2YC1', '2ZCH', '3BGF', '3CX5', '3D85', '3EO1', '3HB3', '3HI1', '3HI6', '3HMX', '3KR3', '3KS0', '3LD8', '3LEV', '3LH2', '3MJ9', '3MXW', '3NGB', '3NH7', '3QWO', '3RKD', '3RU8', '3S37', '3SDY', '3SKJ', '3SO3', '3SQO', '3TT1', '3UC0', '3VG9', '3VI3', '4AEI', '4D9Q', '4DKF', '4DN4', '4DQO', '4DTG', '4DW2', '4ERS', '4F37', '4FFV', '4FQJ', '4G6M', '4HCR', '4HF5', '4HKX', '4HWB', '4I3S', '4JPK', '4K2U', '4KUC', '6WPS']
PDB ID in Testing list (30)
['1FNS', '1N8Z', '1NFD', '1P2C', '1TZH', '1V7M', '2IH3', '2VXT', '3AB0', '3GJF', '3I50', '3LIZ', '3MA9', '3O0R', '3O2D', '3PGF', '3Q1S', '3R1G', '3RAJ', '3RVV', '3T3P', '3U9P', '3ZKM', '4AM0', '4ENE', '4G3Y', '4HJ0', '4HT1', '4I77', '4JR9']

Supplementary Table 7. Performance Evaluation of EpiScan Re-Trained with SOTA Deep Learning methods on **DB1-cdr70**

Methods	*Precision↑*	*Recall↑*	*AUROC↑*	*AUPR↑*	*F1-score↑*	*MCC↑*
PInet (2021)	0.201±0.012	0.613±0.015	0.687±0.015	**0.258±0.005**	0.296±0.019	0.212±0.010
DeepBindPPI (2023)	0.218±0.027	0.562±0.022	0.641±0.012	0.201±0.003	0.256±0.010	0.159±0.003
EpiScan (γ =2)	0.210±0.011	0.695±0.013	0.684±0.018	0.206±0.015	0.286±0.022	0.211±0.013
**EpiScan (γ =1)**	**0.219±0.021**	**0.706±0.025**	**0.701±0.004**	0.244±0.009	**0.306±0.020**	**0.226±0.015**

γ serves as the parameter governing the convolutional kernel in ECA module, which is positively correlated with the size of convolution kernel.

Supplementary Table 8. Performance Evaluation of EpiScan Re-Trained with State-of-the-Art Deep Learning methods on **DB1-cdr70** and Subsequent Testing on **DB2**

Methods	*Precision↑*	*Recall↑*	*AUROC↑*	*AUPR↑*	*F1-score↑*	*MCC↑*
PInet (2021)	0.156	0.635	0.522	0.133	0.201	0.066
DeepBindPPI (2023)	0.192	0.525	0.513	0.106	0.142	0.025
EpiScan (γ =2)	0.201	0.693	0.592	0.176	0.205	0.094
**EpiScan (γ =1)**	**0.212**	**0.695**	**0.623**	**0.201**	**0.229**	**0.134**

Supplementary Table 9. 472 unique samples to serve as an independent test set for the development of production model on the web-server

PDB ID list (472)
['1BJ1', '1BQL', '1CL7', '1DHK', '1DJS', '1DQT', '1DZB', '1EPW', '1EZV', '1FYH', '1G9M', '1G9N', '1HYS', '1K4C', '1M6B', '1MHH', '1NCD', '1OQE', '1OTS', '1OTT', '1QKZ', '1S5H', '1TFX', '1TPX', '1TQC', '1UWX', '1W72', '1Z2C', '1Z92', '1ZWI', '2B4J', '2EC8', '2FJG', '2GHW', '2H32', '2H8P', '2IH1', '2J0O', '2KBH', '2KYH', '2NLJ', '2NXY', '2NXZ', '2NY0', '2NY1', '2NY2', '2NY6', '2P7T', '2QKH', '2VDK', '2W0F', '2XJY', '2YBR', '3BIK', '3CVH', '3DET', '3EJZ', '3H3B', '3HX4', '3JWD', '3LES', '3NIG', '3OR6', '3OR7', '3Q5Y', '3RKC', '3RVV', '3RVW', '3STL', '3STZ', '3THM', '3TJE', '3UX9', '3UYP', '3UZE', '3UZQ', '3UZV', '3V6B', '3VTT', '4E9O', '4HJJ', '4IOI', '4K94', '4KK8', '4KK9', '4LBE', '4LCU', '4LOU', '4M48', '4MA8', '4MSW', '4NFG', '4NIK', '4NZQ', '4NZR', '4P9H', '4QNP', '4TTD', '4UUJ', '4UV7', '4WE2', '4WEB', '4WFE', '4XP9', '4XVU', '4YQX', '4YUE', '4ZFF', '4ZIH', '5A3I', '5AAM', '5C0N', '5D70', '5D71', '5DFW', '5E8D', '5EBL', '5EBM', '5EBW', '5EC1', '5EC2', '5F4J', '5F72', '5FHX', '5GRU', '5GSR', '5GZN', '5HHX', '5HI3', '5HI4', '5HI5', '5IWL', '5J11', '5J9P', '5JHL', '5JYL', '5JYM', '5KAN', '5KN5', '5LDN', '5LX9', '5LXA', '5NGI', '5NIU', '5OCA', '5OGI', '5T29', '5TZT', '5URV', '5VK6', '5VKH', '5VQM', '5VX4', '5W0E', '5W4L', '5WK3', '5X2M', '5X2N', '5X2O', '5X2Q', '6A77', '6ACC', '6AD7', '6ADB', '6AL0', '6BA5', '6BF4', '6BY2', '6BY3', '6CRK', '6CVK', '6DKJ', '6EJG', '6EJM', '6EY5', '6F8P', '6FFY', '6FNZ', '6G8R', '6GKU', '6GLW', '6H2Y', '6H3S', '6HGA', '6HHC', '6HIG', '6HX4', '6HXW', '6I07', '6IAP', '6ICC', '6ID4', '6ION', '6J11', '6J14', '6J15', '6J5F', '6J6Y', '6J71', '6JBT', '6JEP', '6JJP', '6K0Y', '6K65', '6KS0', '6KTR', '6KYZ', '6KZ0', '6LGW', '6LXI', '6LXJ', '6LYN', '6LZ9', '6M0Z', '6M2R', '6M3B', '6M58', '6MFP', '6MG7', '6MTJ', '6MTN', '6MVL', '6N5D', '6N5E', '6N6B', '6N81', '6NFU', '6NFV', '6NHA', '6NMR', '6NMS', '6NMT', '6NMU', '6NMV', '6NYQ', '6O1F', '6O41', '6O9H', '6O9I', '6OAN', '6OC3', '6OC7', '6OGX', '6OHG', '6OKM', '6OOR', '6OSH', '6OTC', '6OY4', '6OZ2', '6P3R', '6P4A', '6P4B', '6P50', '6P67', '6PCU', '6PE8', '6PHB', '6PHC', '6PI7', '6PIS', '6PLK', '6PPG', '6PXH', '6PZE', '6PZF', '6Q0E', '6Q0H', '6Q0I', '6Q0L', '6Q20', '6QB3', '6QB6', '6QIG', '6RLO', '6RP8', '6RPS', '6S3D', '6S5A', '6SUZ', '6SV2', '6SVL', '6TFB', '6TOU', '6TYB', '6U2F', '6U36', '6U38', '6U3I', '6U6U', '6U8C', '6U9S', '6UJ9', '6UMG', '6UMX', '6URH', '6UTE', '6UUH', '6UYD', '6UYF', '6UYM', '6UYN', '6VC9', '6VEL', '6VEP', '6VGR', '6VTW', '6VUG', '6VVU', '6VY4', '6VY6', '6VZI', '6W00', '6W03', '6W7S', '6WAS', '6WGL', '6WH9', '6WHK', '6WIO', '6WIR', '6WIT', '6WIX', '6WM9', '6WMW', '6WO3', '6WO4', '6WO5', '6WOZ', '6WTU', '6WZJ', '6WZK', '6WZL', '6WZM', '6XC3', '6XC4', '6XCJ', '6XKR', '6XLQ', '6XM2', '6XP6', '6XQW', '6XSN', '6XSW', '6XXV', '6XZW', '6Y6C', '6YE3', '6YIO', '6YLA', '6YWC', '6Z2L', '6Z2M', '6ZCZ', '6ZTR', '7AHU', '7B3O', '7BEI', '7BEM', '7BEP', '7BQ5', '7BSC', '7BSD', '7BWJ', '7C61', '7C88', '7CDI', '7CE2', '7CEB', '7CHB', '7CHP', '7CHS', '7CHY', '7CHZ', '7CJ2', '7CM4', '7COE', '7CQC', '7CR5', '7CU5', '7CVT', '7D85', '7DAA', '7DC8', '7DEO', '7DET', '7DFA', '7DFC', '7DHA', '7DJZ', '7DM1', '7DR4', '7DUO', '7E3O', '7E5O', '7E7X', '7E86', '7E8M', '7E9B', '7EAM', '7ELX', '7JIE', '7JKT', '7JMO', '7JN5', '7JOO', '7JTG', '7JTR', '7JUM', '7JX3', '7K93', '7KD6', '7KEO', '7KET', '7KLH', '7KMG', '7KMH', '7KMI', '7KN3', '7KN4', '7KN7', '7KPB', '7KPG', '7KQ7', '7KQG', '7KYL', '7KYO', '7L0N', '7L7D', '7LCV', '7LF7', '7LF8', '7LFA', '7LFB', '7LJ4', '7LJ5', '7LJB', '7LM9', '7LSE', '7LSF', '7LSG', '7LY3', '7M3I', '7MMO', '7MWW', '7MZF', '7MZG', '7MZH', '7MZK', '7N3C', '7N3D', '7N3I', '7N4I', '7N4J', '7NEH', '7NX7', '7NX8', '7NX9', '7NXA', '7NXB', '7O52', '7O9S', '7ORA', '7ORB', '7R6X', '7RAH', '7RCO', '7RK1', '7RK2', '7RKS', '7S0B', '7S13']
PDB ID list : humanized antibody samples with CDR identity below 70% compared to the training data(17)
['5A3I', '5KAN', '5KN5', '6BA5', '60C3', '6P3R', '6VVU', '6W05', '6WZJ', '6WZM', '6XM2', '6XXV', '7D85', '7DC8', '7DM1', '7JIE', '7RCO']

Supplementary Table 10. External data samples for finetuning in web-server model

SARS-CoV2 PDB ID list (14)
['8A94', '8A95', '8CIM', '8D0Z', '8DXS', '8ERR', '8F0G', '8H07', '8H07', '8HC2', '8HHX', '8HHY', '8HHZ', '8J1V']
Flu-HA PDB list (41)
['3GBN','3SDY','3ZTJ','4FP8','4FQJ','4GXU','4HF5','4HFU','4HG4','4HKX','4KVN','4M5Z','4NM8','4R8W','5UGY','4YK4','5JW3','5K9K','5W08','6E3H','6E4X','6NZ7','6Q0O','6Q18','6URM','6XPQ','6XPR','6XPX','6XPY','6XPZ','6XQ2','6XQ4','7MEM','8GV5','4FQK','5IBL','5K9Q','5KAQ','5WKO','6E56','6XQ0']

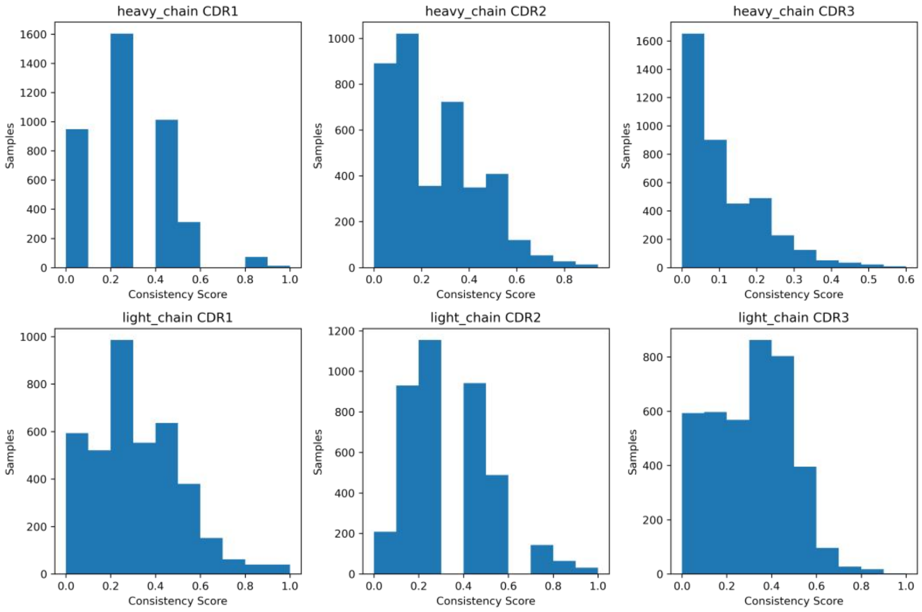

Supplementary Fig. 1. Illustrates the consistency scores of heavy and light chains across the CDR1, CDR2, and CDR3 regions. The average consistency scores for the heavy chain are 0.248, 0.255, and 0.101 for CDR1, CDR2, and CDR3, respectively. For the light chain, the average consistency scores are 0.320, 0.337, and 0.295 for the corresponding regions. These graphs clearly depict the sample sizes for each region at different consistency score levels.

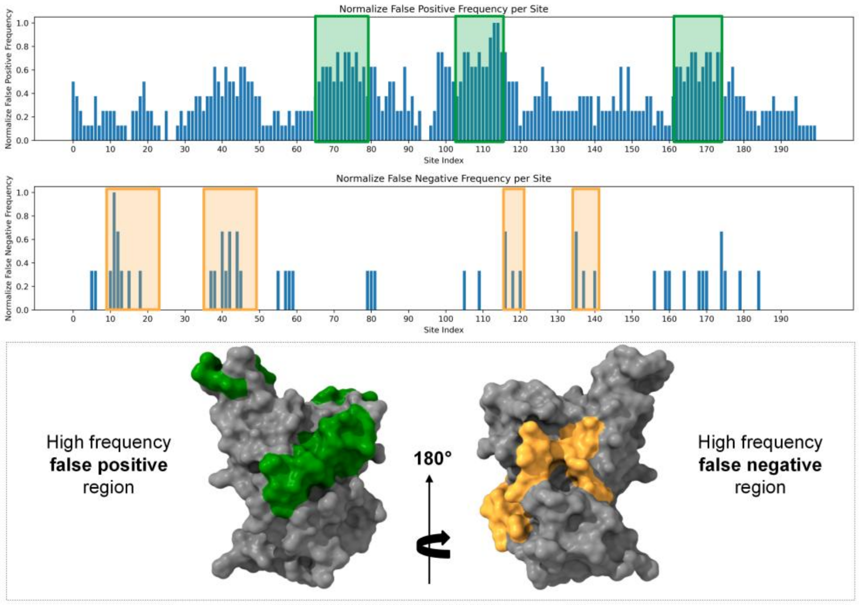

Supplementary Fig. 2. A visual representation of high-frequency false positive and negative regions on a protein structure. The accompanying bar graphs depict EpiScan DB2 test results for each residue within the Receptor-Binding Domain (RBD).

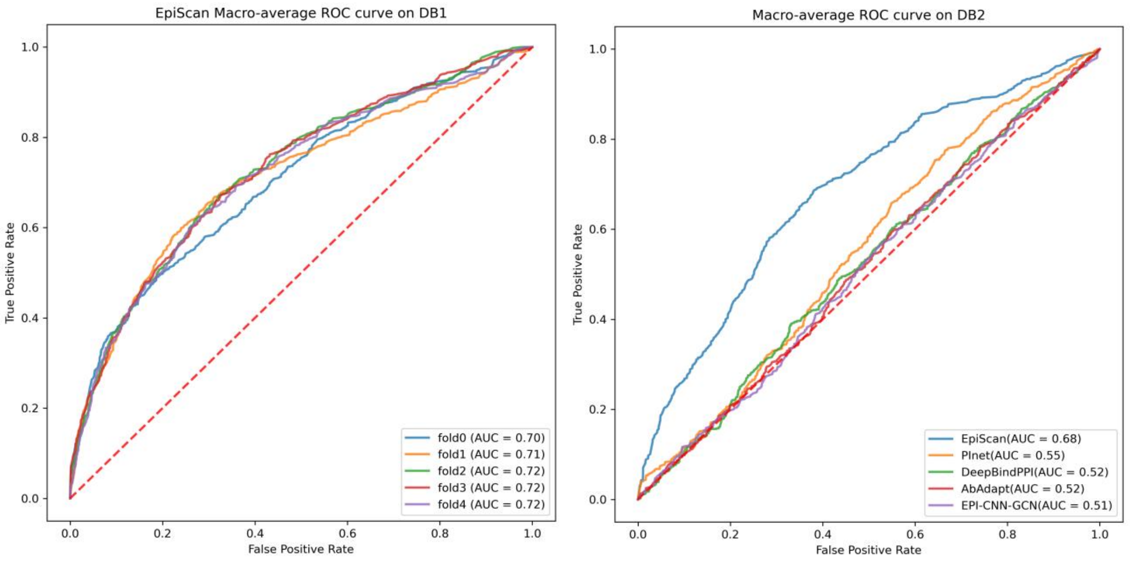

Supplementary Fig. 3. Macro-average Receiver Operating Characteristic (ROC) curves for epitope prediction models on Databases 1 (DB1) and 2 (DB2). For DB1, four-fold cross-validation results are shown with fold-specific area under the curve (AUC) values: fold0 (AUC = 0.70), fold1 (AUC = 0.71), fold2 (AUC = 0.72), fold3 (AUC = 0.72), and fold4 (AUC = 0.72). The dashed line represents a random classifier's expected performance. For DB2, the AUC values for different prediction models are depicted: EpiScan (AUC = 0.68), BinepiTCR (AUC = 0.56), DeepBindRPI (AUC = 0.52), and Epi-CNN-GCN (AUC = 0.51).

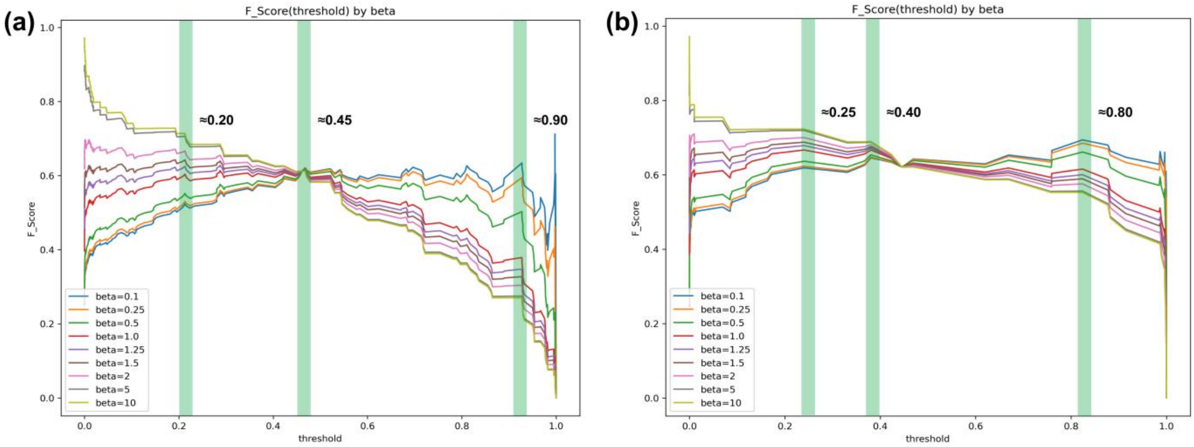

Supplementary Fig. 4. Calculating F_score with different values of β. **(a)** plotting the results on SARS-CoV-2 testing set. **(b)** plotting the results on SARS-CoV testing set. Thresholds favoring *precision* are primarily recommended based on β values of 0.25 and 0.5, while those favoring *recall* are recommended based on β values of 1.25, 1.5, and 2. The cases of β values at 0.1, 5, and 10 are relatively extreme and are provided for users as exceptional references, where thresholds can be determined at the corresponding peak values of the curves.

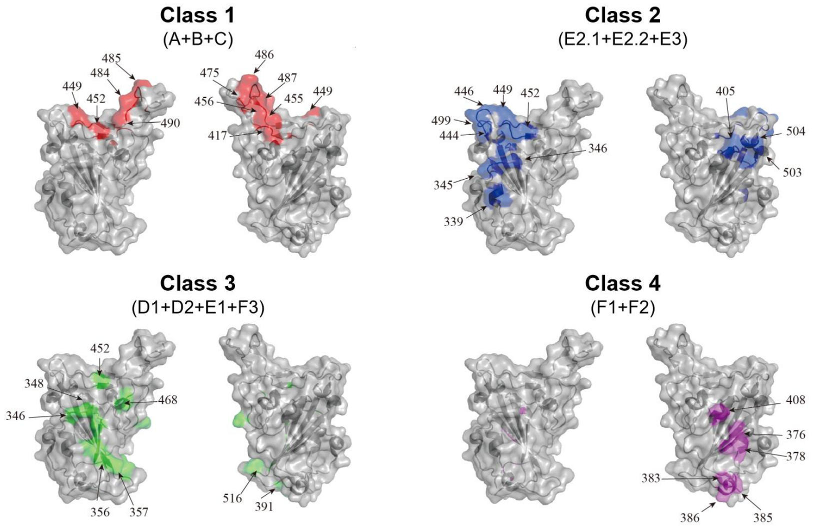

Supplementary Fig. 5. Illustration of the 12 DMS epitope groups as described by Cao et al., categorized into four non-overlapping structural classes. Representations for each class are as follows: Class 1 includes binding groups A, B, and C; Class 2 includes D1, D2, E1, and F3; Class 3 includes E2.1, E2.2, and E3; Class 4 includes F1 and F2. The highlighted regions depict the specific binding interactions for each class, providing a visual delineation of the spatial arrangement and clustering of the binding sites on the molecular surface.
